# 3D modelling and simulation of the dispersion of droplets and drops carrying the SARS-CoV-2 virus in a railway transport coach

**DOI:** 10.1038/s41598-022-08067-6

**Published:** 2022-03-07

**Authors:** Patrick Armand, Jérémie Tâche

**Affiliations:** 1 CEA, DAM, DIF, 91297 Arpajon, France; 2 FLUIDIAN, 95000 Cergy, France

**Keywords:** Health care, Engineering, Mathematics and computing

## Abstract

Computational fluid dynamics (CFD) modelling and 3D simulations of the air flow and dispersion of droplets or drops in semi-confined ventilated spaces have found topical applications with the unfortunate development of the Covid-19 pandemic. As an illustration of this scenario, we have considered the specific situation of a railroad coach containing a seated passenger infected with the SARS-CoV-2 virus (and not wearing a face mask) who, by breathing and coughing, releases droplets and drops that contain the virus and that present aerodynamic diameters between 1 and 1000 µm. The air flow is generated by the ventilation in the rail coach. While essentially 3D, the flow is directed from the bottom to the top of the carriage and comprises large to small eddies visualised by means of streamlines. The space and time distribution of the droplets and drops is computed using both an Eulerian model and a Lagrangian model. The results of the two modelling approaches are fully consistent and clearly illustrate the different behaviours of the drops, which fall down close to the infected passenger, and the droplets, which are carried along with the air flow and invade a large portion of the rail coach. This outcome is physically sound and demonstrates the relevance of CFD for simulating the transport and dispersion of droplets and drops with any diameter in enclosed ventilated spaces. As coughing produces drops and breathing produces droplets, both modes of transmission of the SARS-CoV-2 virus in human secretions have been accounted for in our 3D numerical study. Beyond the specific, practical application of the rail coach, this study offers a much broader scope by demonstrating the feasibility and usefulness of 3D numerical simulations based on CFD. As a matter of fact, the same computational approach that has been implemented in our study can be applied to a huge variety of ventilated indoor environments such as restaurants, performance halls, classrooms and open-plan offices in order to evaluate if their occupation could be critical with respect to the transmission of the SARS-CoV-2 virus or to other airborne respiratory infectious agents, thereby enabling relevant recommendations to be made.

## Introduction

Since late 2019, the disease referred to as Covid-19 has disrupted human life and activities and generated a crisis of planetary dimensions. The Covid-19 outbreak has provoked numerous controversial debates about how to slow or stop the propagation of the epidemic throughout the population. The infectious agent of the disease is the SARS-CoV-2 virus, which penetrates into the respiratory tract. Its modes of transmission have long been discussed. At the outset of the epidemic, touching unclean objects (also known as “fomites”) with the hands was considered to be the prevalent mode of contamination, and the virus was assumed to be transported on the hands. Starting from spring 2020, however, an increasingly large part of the international medical community has suggested that the virus could also be airborne and transmitted in the air through the droplets produced by infected people. For instance, according to one of the first studies performed in the Chinese province of Wuhan, the cradle of the pandemic, and published on 11 April 2020 by the US Center for Disease Control and Prevention (CDC), the virus can travel a distance of up to 4 m from a sick person^1^. After this, on 7 July 2020, a group of 239 scientists published a collective letter to the World Health Organization (WHO) indicating that there was a risk of aerial transmission of SARS-CoV-2 and prescribing supplementary precautionary measures^2^. Subsequently, in a press release on 8 July 2020, the WHO announced that there was evidence of Covid-19 airway transmission^3^.

Following W. F. Wells’s historic work on tuberculosis in the 1930’s, the host-to-host transmission of respiratory diseases has been regarded by the WHO and by agencies such as the CDC to be dependent on the size of the droplets emitted, with various diameter cut-offs ranging from 5 to 10 μm. The MIT Bourouiba Research Group on Covid-19^4^, however, noticed that such a dichotomy may not reflect what actually occurs with respiratory emissions. Researchers at the US National Institutes of Health^5,6^ carried out experiments showing that during speech, a person can produce more than 1000 droplets per minute, with the smallest droplets of up to 20 µm in diameter remaining suspended in the air for 10 min, and the bulkier drops crashing almost instantly onto accessible surfaces. This was confirmed in a review paper^7^ and in publications gathering data about drops and droplets produced in human secretions, as synthesised hereafter. The SARS-CoV-2 virus has a diameter of 70–90 nm^8^ and may be carried by drops and droplets^9,10^. While drops of at least 100 µm in diameter reach the ground within 1 s without significant evaporation^11^, smaller droplets fall more slowly and evaporate more rapidly^12–15^. The spittle produced by individuals is often categorised as drops of around 10 µm up to 1 mm in diameter, with the largest of these presenting ballistic trajectories, or as droplets of less than 5 µm (and desiccated droplet nuclei) known as aerosols, which remain airborne in the range of minutes to hours^9–17^. While talking, a person ejects tens of small particles per second with diameters between 0.1 µm and 1 mm^18^ and with a speed of the order of 1 m s^−1^ ^**19**^. This is the most common source of aerosol that can be inhaled by other people^19–21^. While coughing leads to the ejection of 100–1000 fluid particles per second with a speed of around 10 m s^−1^, sneezing generates 1000–10,000 fluid particles per second with a speed of up to 20 m s^−1^. The droplets can travel distances of 7 to 8 m depending on their initial velocity and the ambient air flow^7^. Their fate is also influenced by the temperature and humidity of the ambient air. Thus, the “lifetime” of a droplet ranges from seconds to minutes, with moist, warm air tending to extend this duration^22^. After evaporation, the nuclei of the droplets may remain suspended for hours, and remain virulent for more than three hours^9^.

While the knowledge regarding the transmission routes and behaviour of the SARS-CoV-2 virus is still evolving at the time of this writing, certain facts can be established from the previous review:

- Apart from infection due to a direct contact with a previously contaminated surface, coughs, sneezes, respiration and speech are events during which individuals close to each other may become contaminated;
- Interpersonal transmission of Covid-19 may happen by means of respiratory droplets and spittle produced when coughing or sneezing;
- Some studies have proved that the SARS-CoV-2 virus is present in drops with large diameters of about 100 µm to 1 mm, which are projected from one individual to another;
- There are also several studies pointing out the risk of transmission through aerosols composed of droplets with diameters of about 1 µm to 10 µm.

As for any kind of aerial pollution, the transport and dispersion of drops and droplets containing viruses are strongly influenced by the air flow, both outdoors in the atmospheric environment and indoors in any semi-confined spaces. In the latter case, knowledge of the ventilation features is crucial for determining the space and time distribution of the virus, whether it is SARS-CoV-2 or any other airborne infectious agent. Moreover, ventilation certainly plays a major role in controlling and limiting the propagation of infectious diseases. To this effect, the American Society of Heating, Refrigerating and Air‑Conditioning Engineers (ASHRAE) declared in April 2020 that “the transmission of SARS-CoV-2 through the air was sufficiently likely that airborne exposure to the virus should be controlled (…) including the operation of heating, ventilating, and air-conditioning systems (…) in order to reduce airborne exposures”^23^**.**

In this perspective, the mode of transmission of the SARS-Cov-2 virus, which relies on droplets transported and dispersed though the atmospheric environment and indoor spaces, at distances from less than one meter to a few meters (and perhaps more), opens up possibilities for using computational fluid dynamics (CFD). Generally speaking, CFD enables choices to be made regarding efficient designs and developments in order to reach desired results and to prevent or limit the adverse effects of critical situations. Therefore, CFD could find a specific breakthrough application in response to the propagation of infectious agents in enclosed spaces. This is precisely the objective of this paper, which aims to demonstrate the relevance of physical modelling and numerical simulation using tried and tested CFD computer software to evaluate the transport and dispersion of drops and droplets carrying the SARS-CoV-2 virus in human secretions and, at a later stage, to evaluate the health consequences of the virus. In addition, the 3D numerical study intends to compare the Eulerian and Lagrangian approaches for dispersion modelling. It is thus a matter of determining which methods are appropriate for simulating the transport and dispersion of drops and droplets in an indoor environment. Subsequently, these methods will be available to be re-employed with confidence in semi-enclosed ventilated spaces of different geometries.

While CFD certainly has strong potential to provide a better understanding of the complex, non-intuitive distribution pattern of droplets expectorated by infected humans in semi-confined spaces, even now only a few studies have so far applied CFD to the dispersion of virus-laden aerosols. Without being exhaustive, a review of some of these studies is given hereafter. In all of these studies except for the last one, the individuals releasing the particles carrying the virus were not wearing a mask.

Wang et al.^24^ studied the near-field fate of liquid particles between 1 and 100 µm produced during sneezing. To do this, they implemented a hybrid Eulerian–Lagrangian numerical approach of the flow and particles and took into account the evaporation of the particles or their condensation in air initially at rest. The authors pointed out the significant influence of ambient conditions of temperature and relative humidity on the evaporation. At low temperature and high humidity, particles down to a few tens of microns evaporated very slowly and remained in suspension. Dbouk and Drikakis^25^ were interested in the distance traveled by the drops emitted during a coughing episode for different wind speeds of 0, 4 and 15 km h^−1^. They relied on experiments to use a Weibull distribution of the drops (of 80 µm average diameter) at the exit of a mouth whose shape was modelled. The authors solved the equations of fluid dynamics in the RANS formalism with the k-omega turbulence model. The dispersion of the drops was evaluated by a Lagrangian method, taking into account the phase changes of the drops and their interactions. The simulations were carried out with the Open FOAM software. The authors showed that the drops were still suspended 6 m beyond their point of origin when carried by the strongest flow. In another study, Dbouk and Drikakis^26^ applied the same turbulence modelling of the flow and dispersion of drops emitted by a simplistic humanoid manikin in an elevator cabin. As expected, the distribution of the drops strongly depended on how the elevator cabin was ventilated. All these studies are informative because they account for the evaporation of liquid particles. However, in the first two of them, neither humans, nor their influence on the flow are modelled. In addition, the length of the simulation domain is limited to 1 m or 6 m while, under certain conditions, the drops continue their progression in the air well beyond these distances. In these articles, no real environment, even simplified, is considered unlike in the following papers.

Vuorinen et al.^27^ made use of large-eddy simulation (LES) models to simulate the transport and dispersion of particles from 1 to 20 µm in a supermarket reduced to four rows of shelves populated in between with two coughing manikins. PALM, Open FOAM and FDS models were used to model the shelves with crude simplifications. The dispersion modelling in PALM was Lagrangian, while it was Eulerian within the other models. One can notice that the geometry, the human occupancy and the ventilation are oversimplified. Moreover, Vuorinen et al. do not leverage the capabilities of LES simulations, as they performed only one execution of the computations with FDS and ten executions with Open FOAM and PALM. They do not account for particles larger than 20 µm on the grounds that the drying process of larger particles should be rapid. Contrariwise, we argue that particles of all sizes should be considered for the sake of exhaustiveness and that their trajectories and distributions should be simulated using a suitable CFD model. Vuorinen et al. conclude that the “safety distance” should be 4 m, which certainly does not constitute an overall result, but is extremely dependent on the flow conditions, the human source of emission, and the size of the particles expectorated.

Abuhegazy et al.^28^ made use of the ANSYS FLUENT software package to simulate the distribution and deposition of particles from 1 to 50 µm in a classroom equipped with air conditioning. One student was assumed to emit particles, with no mention being made regarding the duration of the release. The flow simulations were carried out with an RNG k-epsilon model. The trajectories of the particles were computed with a Lagrangian model. The particles with diameters of less than 15 µm mostly leave the room through the extraction vents, while a large proportion of the particles with diameters greater than 20 µm settle on the accessible surfaces, including glass barriers. One can notice that the results acutely depend on the shape of the obstacles, which include the individuals, who are extremely simplified. Adwibowo^29^ also considered the dispersion of micrometric particles in an imitation classroom using a Lattice-Boltzmann model. The aim of the simulations was to assess if protective shields reduce one’s vulnerability to droplets emitted when breathing. Unfortunately, no manikins were used to mimic any individuals present.

Qian and Li^30^ explored the influence of ventilation on the removal of particles from 1 to 50 µm in diameter generated by patients breathing in a common room of a hospital. The flow simulations were performed with an RNG k-epsilon model, and the dispersion was evaluated with a Lagrangian model. While the larger particles were almost insensitive to the configuration of the ventilation due to their tendency to settle very quickly, the distribution of the small particles was affected by the location of the inlet and outlet vents. Unfortunately, the geometry and the ventilation system considered in this study seem to be very specific, and it is unclear if the patients were actually modelled. Wang et al.^31^ also dealt with the optimisation of air distribution in a hospital ward for minimising cross-infection among patients. Simulations were performed with ANSYS FLUENT software using a realisable k-epsilon flow model and a Lagrangian dispersion model. Again, the patients were represented in a very simplified way (as parallelepiped solids), thereby limiting the reliability of the computations. Crawford et al.^32^ were interested in the distribution of micrometric particles emitted during the coughing and breathing of patients placed in intensive care units and equipped with oxygenation devices. The study used Power Flow® CFD software which is based on the Lattice Boltzmann method to simulate the flow and on motion tracking of the droplets. The authors showed that an adequate orientation of the bed in relation to the ventilation system and an additional air handling unit can increase the number of particles extracted and decrease the number of particles deposited. The study is relatively generic and could be adapted to different hospital room configurations. However, the authors admit that due to the complexity of the model, the simulated time period of 45 s is very short and the geometry of the room as well as the patient are extremely simplified.

Desai et al.^33^ made use of the ANSYS FLUENT software package to assess the risk of SARS-CoV-2 transmission in intercontinental commercial aircraft. The flow was simulated in 2D cross-sections of Boeing and Airbus airplanes. The dispersion of particles emitted by passengers was computed using the Eulerian approach. The discussion focuses on the configurations of the seats and whether or not they favour the propagation of the virus. Unfortunately, the study is limited to 2D simulations, while the flow and dispersion in the cabins of the planes are most likely 3D. Moreover, only the seats and not the passengers are modelled. Mathai et al.^34^ emphasized that a passenger car cabin was a high risk space for the transmission of pathogens. Like the previous authors, they used ANSYS FLUENT to solve the Navier–Stokes equations in the Reynolds formalism with k-epsilon turbulence model. The authors studied the transmission of droplets from the driver to his or her rear passenger, one of the two occupants being contaminated, for different opening configurations of the vehicle's windows. The flows were very different depending on the case and strongly influenced the distribution of the droplets. Although the study is interesting, the digital mock-up is greatly simplified. Thus, the interior of the car is not modelled at all and the occupants are represented by cylinders. Jayaweera et al.^35^ examined the transmission of particles of different sizes expectorated by individuals when coughing. They considered an infected person in a plane, or at the rear of a car, or in a healthcare centre. The authors presented the streamlines departing from the mouth of the person in these different situations. Unfortunately, it is unclear if the streamlines were issued by CFD computations or if they were a qualitative representation of what the flow could be. Moreover, the expectorating person ought to be wearing a surgical mask or a respirator, but again there is no information about the modelling of the mask.

In summary, while these studies represent valuable efforts in modelling and simulation, they are impaired by a number of limitations in terms of digital mock-up realism of the modelled confined space and, principally, of the human beings populating this space, and in terms of size range completeness of the particles accounted for. In contrast, we have opted to pay careful attention to the realism of the geometry, including the human beings, and to consider virus-laden particles ranging in size over four orders of magnitude. The case study we have developed herein corresponds to a public railway transport coach in which a passenger infected with the SARS-CoV-2 virus is seated and possibly contaminates other travellers. To begin with, the passenger wears no face mask and produces drops and droplets when coughing and breathing.

With regard to particle size, it is important to recall the usual terminology. By definition, an “aerosol” designates solid or liquid particles suspended in the air. The droplets satisfying this criterion have an aerodynamic diameter in the range between some tenths of micrometers (µm) to some micrometers. The aerodynamic diameter gives weighting to the geometric diameter according to the density (considered equal to 1 in our computations). Particles with aerodynamic diameters above some micrometers do not form an aerosol. By convention, we make use of the word “droplets” for particles whose diameter is between 1 and 10 µm, whereas the word “drops” is used for particles with a diameter between 100 and 1000 µm.

The following parts of the paper are dedicated to presenting the CFD study with the objective of proving the capability of the numerical modelling to represent the air flow and the transport and dispersion of drops and droplets in semi-enclosed ventilated spaces. A railway coach of the type used by millions worldwide for travel on suburban transportation networks is taken as an example in our methodology. The results of this case study are useful not only for similar situations, but can also be directly transposed to any other semi-confined ventilated place, as discussed later on. A summary of the results regarding the airflow and the dispersion of drops and droplets is proposed hereafter. These results lay the foundation for a discussion about the findings of the study, whose methods are presented in the final part of the paper.

## Results

### Design of a 3D digital mock-up of a populated transport carriage

Regional railway transportation networks all around the world put millions of commuters into contact with each other every day. Thus, there is a strong interest in studying how airborne viruses, SARS-CoV-2 among others, can propagate in the semi-confined ventilated internal space of a carriage when the viruses are emitted by the passengers in the form of drops or droplets.

While there exists a wide variety of manufacturers and models of rail coaches, we opted to use a generic carriage with no protruding features. It should be recalled that our objective is to demonstrate the feasibility of the 3D numerical study without designating a particular rail coach whose configuration is specifically conducive to disseminating viruses or to preventing their dissemination. Indeed, our study can be adapted to any kind of train carriage, and modellers wishing to implement such work could engage with their local or national railway companies to obtain information about the specific rail coaches operated in their cities or countries.

**Figure 1:**
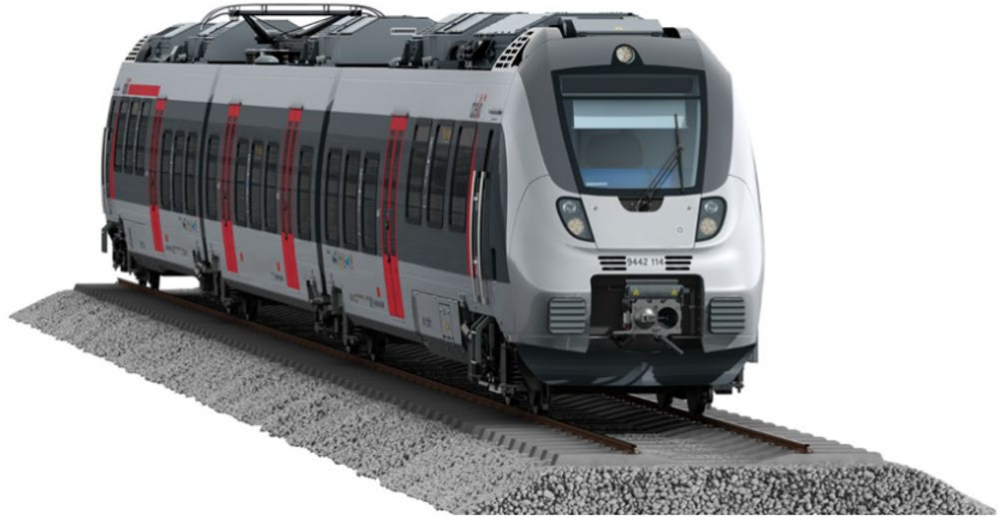
Model of rail coach chosen for the CFD numerical study.

**Figure 2:**
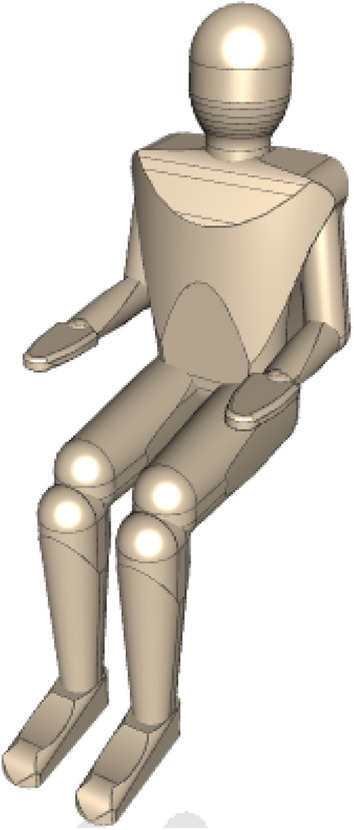
Representation of the reference human manikin used as a train passenger.

Figure 1 shows the single-level rail coach taken as an example throughout the numerical study. The dimensions of the carriage are 15.5 m in length, 2.5 m in width and 2.6 m in height. The original high-precision 3D data of the rail coach geometry and internal layout have been processed from the https://www.turbosquid.com site and transformed in order to generate a 3D mesh for CFD computations. The coach is occupied by passengers represented by humanoid manikins selected from the https://www.traceparts.com site and shown in Fig. 2.

The level of detail of the humanoids is intermediate, situated between oversimplified individuals and overly detailed manikins that could complicate and uselessly prolong the computations. All manikins have a standing size of 1.75 m, and by choice of the modellers, they are integrated into the rail coach in seated positions. Of course, other configurations with manikins of different sizes corresponding to male and female adults, children or infants, and with a combination of standing and seated manikins, could have been examined.

**Figure 3:**
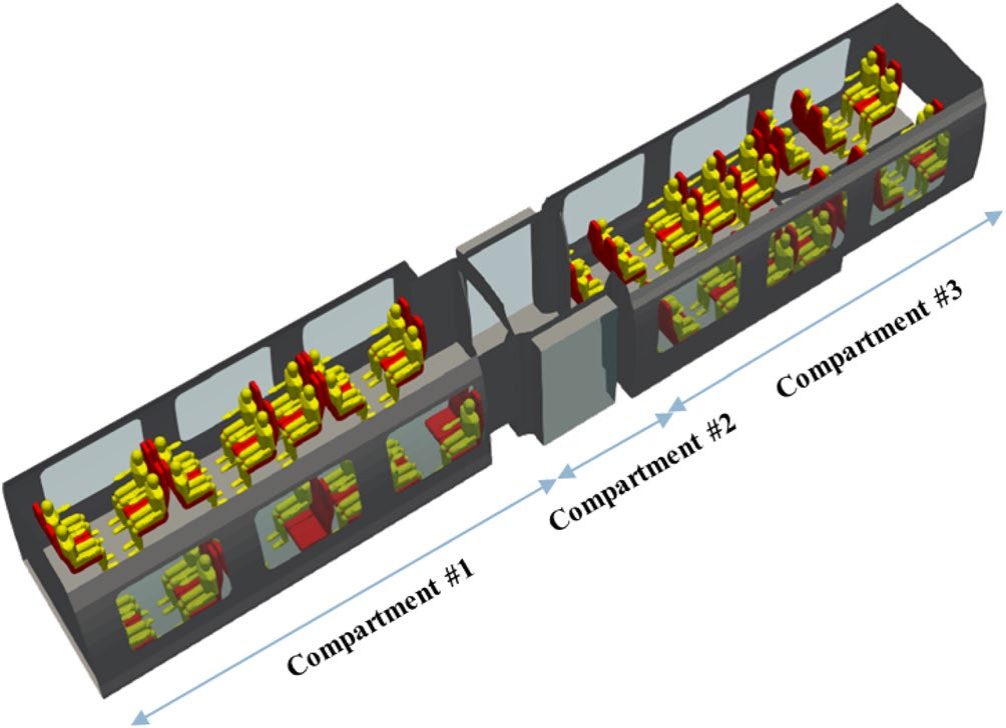
Representation of the mock-up combining the internal space of the rail coach and the passengers.

Figure 3 illustrates the geometry of the rail coach occupied by the passengers. The carriage is subdivided into compartments 1 and 3, which are occupied by the passengers, and compartment 2, from which people get into and out of the train. In the study, the occupation rates of compartments 1 and 3 by seated passengers are respectively 92% and 100%. These figures chosen by the modellers could be varied to examine the influence of the rail coach occupation rate on the distribution of the drops and droplets secreted by the passengers.

**Figure 4:**
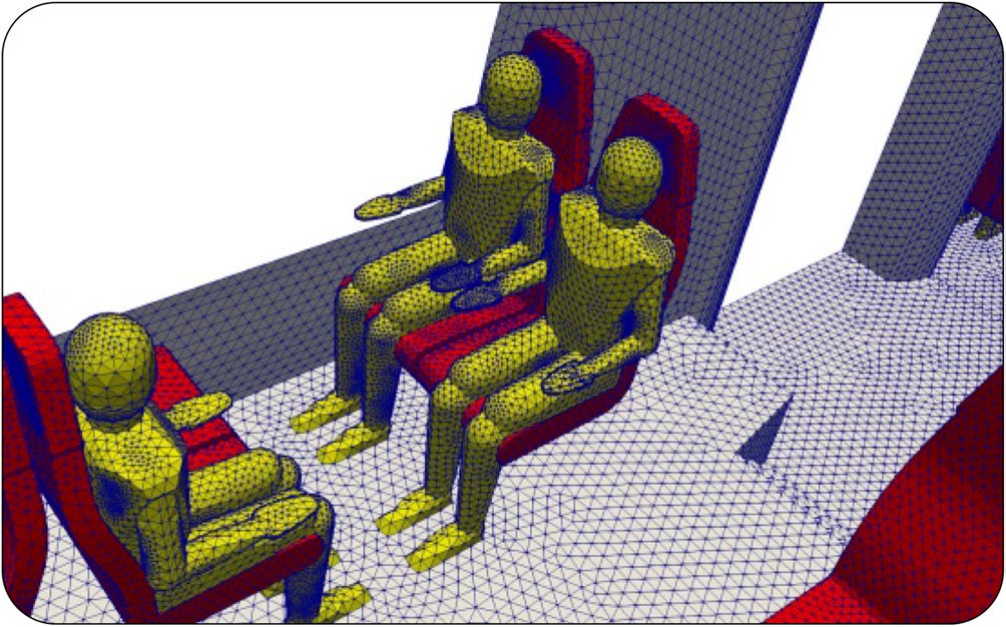
Zoom on the triangular surface mesh of the internal walls of the rail coach and three manikins.

An unstructured CFD mesh was generated from the geometry with tetrahedral cells in order to fit the complex internal geometry of the train and of the passengers, as can be observed on Fig. 4. The minimum cell size is 1 cm near the mouth of the manikins, and 3 cm on the body of the manikins and on the seats. The maximum size of the cells is between 5 and 10 cm on the internal walls of the rail coach. The mesh consists of 4 million cells in total, a number that was proved to satisfy the convergence of the flow field as shown in the “Methods” section.

### Simulation of the air flow in the rail coach and around the passengers

In order to model the air flow in the carriage, one has to implement a ventilation system as with any other semi-confined space. The ventilation in the mock-up of the carriage is operated in the same way as for an actual rail coach. It is described briefly hereafter and in more detail in the “Methods” section. While the ventilation system considered in the mock-up of the carriage is quite common, it may be different in other rail coaches. Still, it would not be a major issue to take account of alternative blowing and extracting air vents corresponding to different models of carriages.

The ventilation system is organised by zones corresponding to the volumes defined in Fig. 3. We assume that only fresh air is supplied from the outside of each end of the carriage with imposed velocities and flow rates. Air extraction is performed through slits in the roof of each volume of the carriage. In the volumes 1 and 3 occupied by the passengers, the exit velocities and flow rates are imposed, while in the central volume 2, air is extracted at atmospheric pressure, resulting in a balance of air flows between the volumes. With these characteristics, the ventilation of the rail coach is efficient and involves the entire space of the carriage. The air flow is directed globally from the bottom to the top of the carriage. The renewal rate of the air is 8.7 or, in other words, the air is refreshed every 7 min. The velocities of the supplied and extracted air are less than 0.2 m s^−1^, corresponding to the soft ventilation prescribed by the railway operators to ensure comfortable conditions for the passengers.

Translating the ventilation features into inlet and outlet boundary conditions enabled air flow simulations to be carried out. First, the stationary solution of the 3D turbulent flow in the rail coach was computed to initialise the air flow. Then, transient computations of the flow were performed to account for the coughing or breathing of passengers. The CFD tool used for the study is referred to as Code_SATURNE. It is described in the “Methods” section, as are the boundary conditions for the modelling of the air flow and the turbulence.

### Results of the 3D stationary air flow simulations

The results presented here are those regarding the stationary solution of the 3D flow simulation in the ventilated rail coach in the presence of passengers.

**Figure 5:**
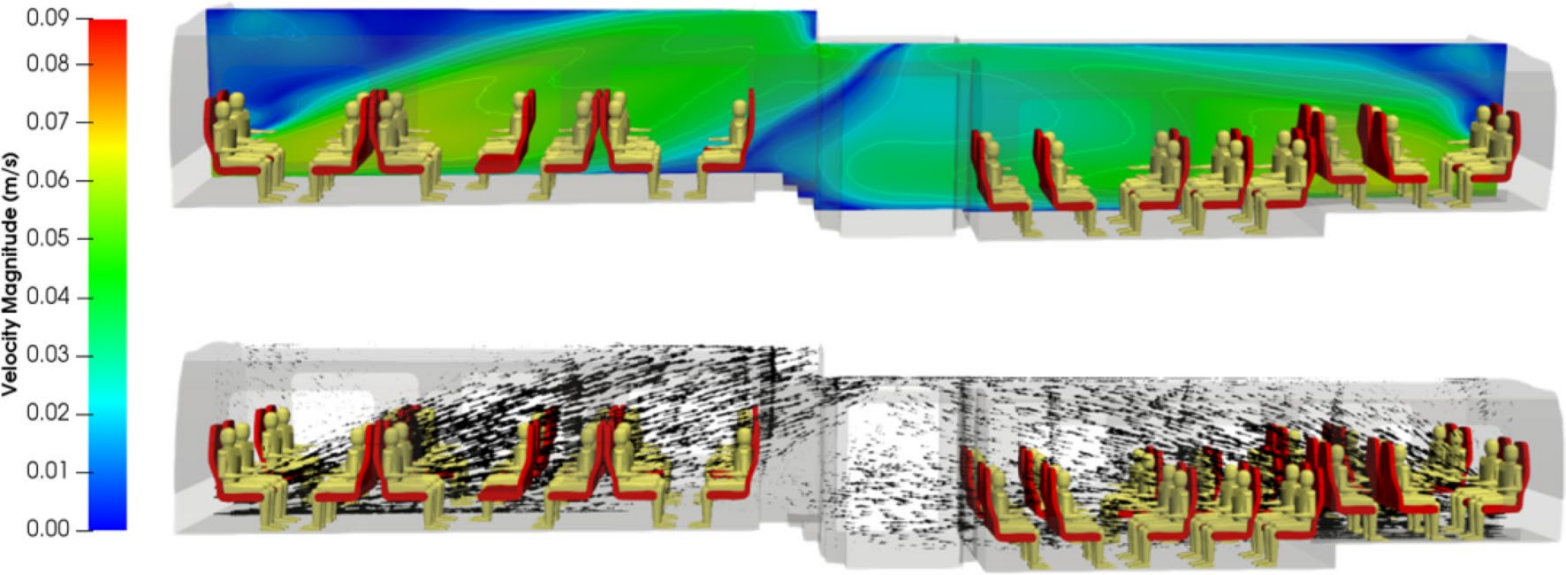
Velocity magnitude (in m s^−1^) and velocity vectors in a vertical plane across the middle of the carriage.

**Figure 6:**
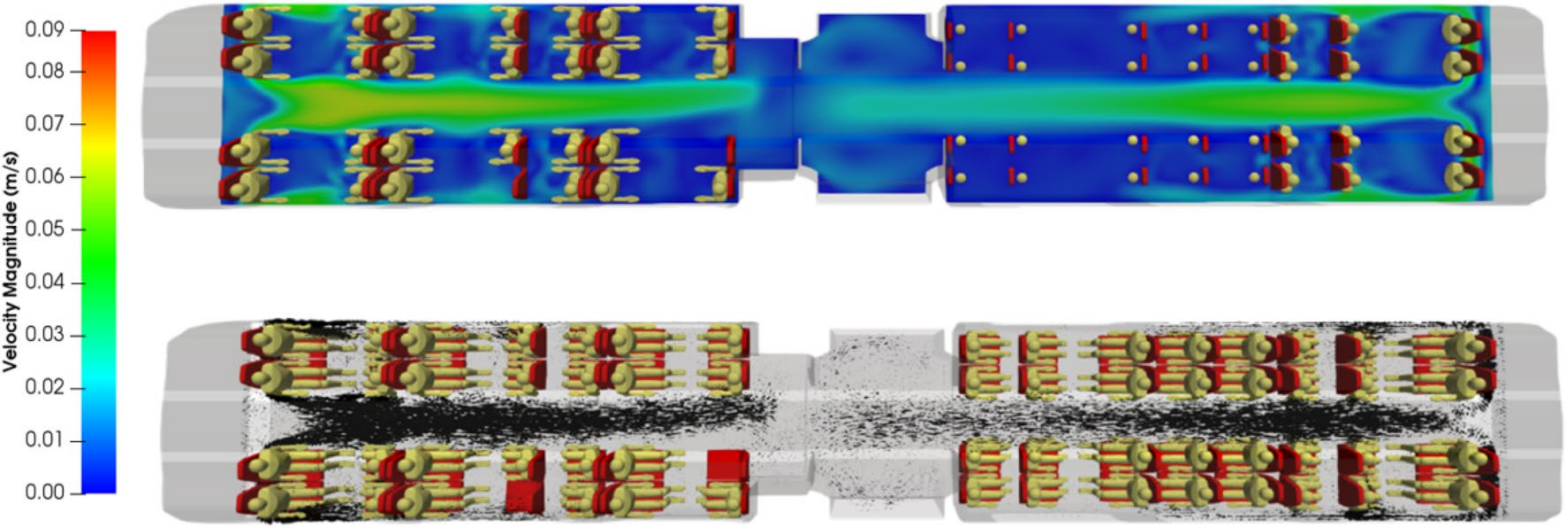
Velocity magnitude (in m s^−1^) and velocity vectors in a horizontal plane at 1 m above the floor in compartment 1 of the carriage.

**Figure 7:**
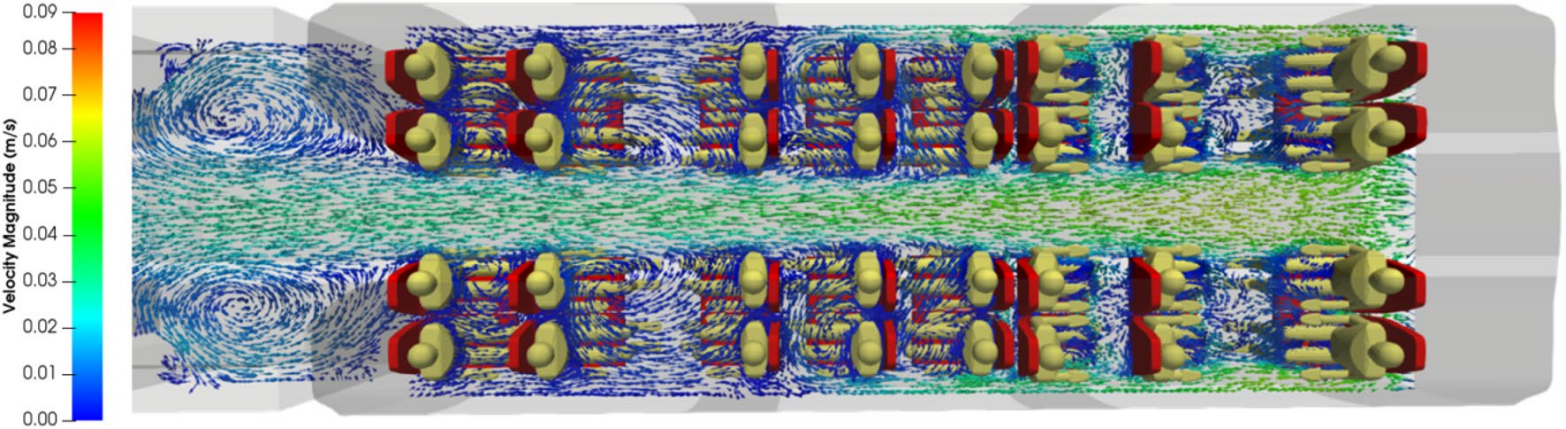
Zoom on the velocity vectors coloured by the velocity magnitude (in m s^−1^) in a horizontal plane at 1 m above the floor in compartment 3 of the carriage.

Figures 5 and 6 respectively show the velocity magnitude and vectors in a vertical cross-section and a horizontal-cross section of the carriage. Figure 7 illustrates the velocity vectors coloured by the velocity magnitude in a horizontal cross-section of the carriage. As expected from the ventilation system considered, the air flows from the ends of the rail coach to its middle and from the bottom to the top of the coach. The velocity is low: it is less than 7 cm s^−1^ everywhere. The central aisle in compartments 1 and 3 of the carriage (see Fig. 3) is unobstructed and channels the flow. This is where the highest velocities in the carriage occur. On the contrary, seats and passengers inside the carriage represent obstacles; between them, eddies are formed, both horizontally and vertically. By this combination, multiple 3D local, low-velocity recirculations between the passengers are superimposed upon the principal air flow from the blowing vents at the end of compartments 1 and 3 to the extracting slits distributed all along the roof of the carriage. Finally, the changes in the sections between compartments 1 and 2 and compartments 3 and 2 of the rail coach also lead to the development of vortices.

**Figure 8:**
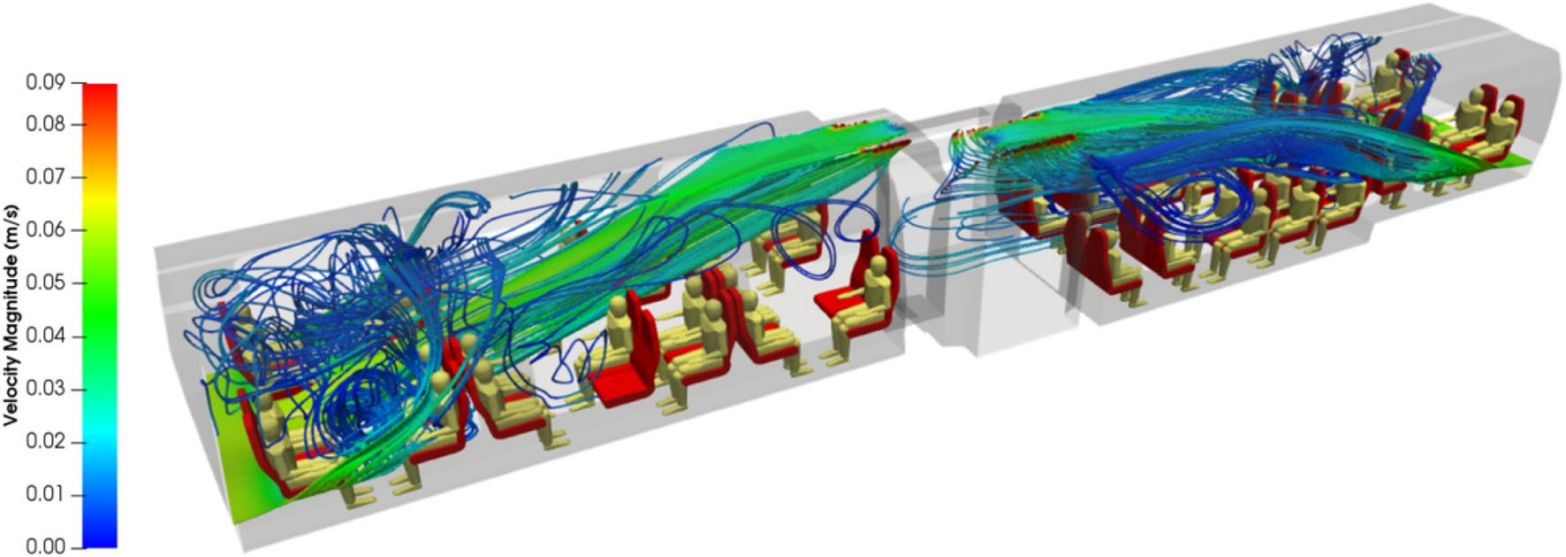
Oblique view of the streamlines departing from the blowing vents at the ends of the carriage and coloured by the velocity magnitude.

**Figure 9:**
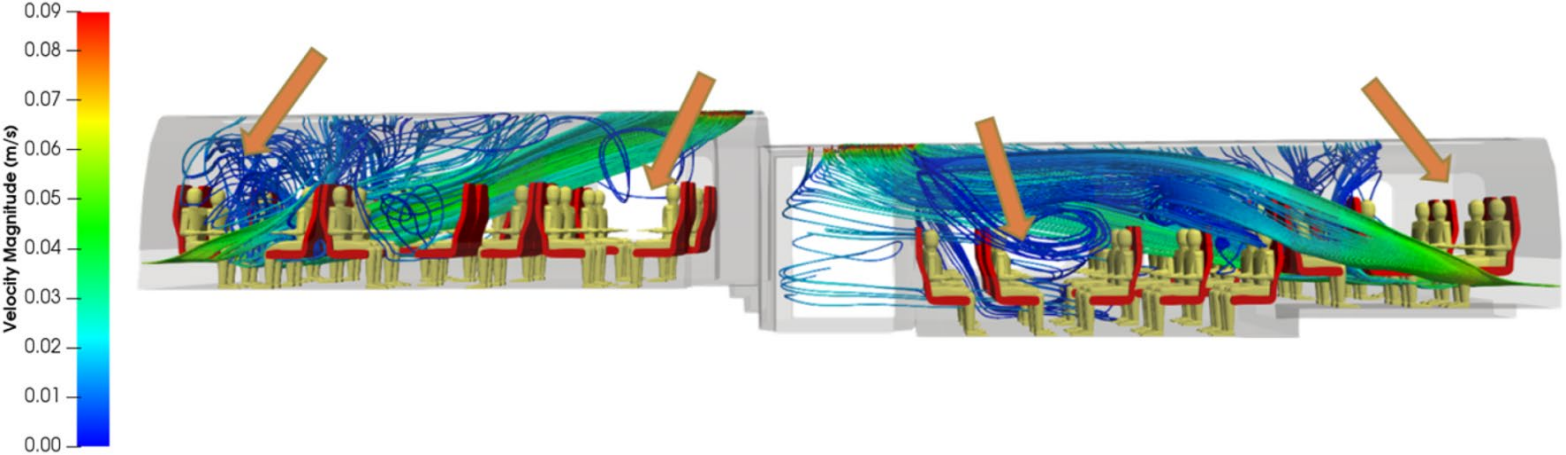
Side view of the streamlines departing from the blowing vents at the ends of the carriage and coloured by the velocity magnitude.

Figures 8 and 9 show streamlines of the air flow coming from the blowing vents at the ends of the carriage. From a global point of view, streamlines depart from the endpoints of the rail coach in compartments 1 and 3, move from the bottom to the top of the carriage, and join the extracting vents in the roof of compartments 1, 2 and 3 of the rail coach. In more detail, streamlines issued from close points may exhibit very different routes as the inner space of the carriage comprises an unobstructed aisle and obstacles constituted by the seats and the passengers. While several streamlines take direct routes along the aisle, many others follow 3D eddies developing between the passengers seated face-to-face or between the rows of seats. The local recirculations are characterised by low or even very low velocities, and some spaces are quite unventilated as indicated by the orange arrows in Fig. 9.

The next step of the numerical study involved the unsteady computations of the air flow and dispersion of the drops and droplets emitted by passengers when coughing or breathing. The 3D air velocity field in the rail coach is disturbed only very locally by the initial velocity of the drops and droplets. Thus, it is not presented in this paper, and the next sub-sections focus on the source terms generated by the coughing and breathing and on the simulation of the space and time distributions of the drops and droplets in the inner space of the rail coach.

### Simulation of dissemination events in the rail coach

**Figure 10:**
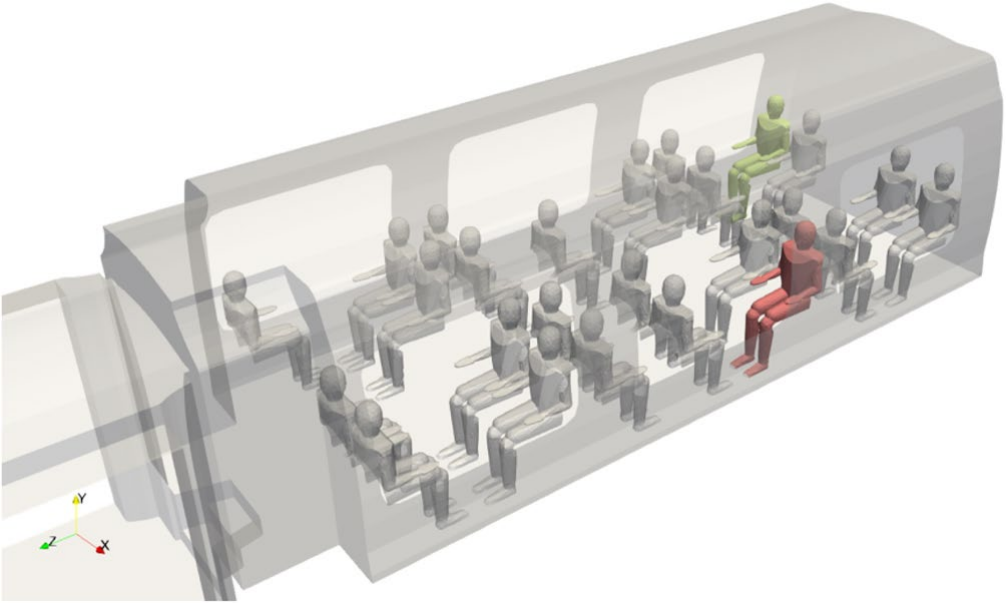
Alternative locations of the coughing passenger (in red and in green) and of the breathing passenger (in green) assumed to be infected with the virus. The manikins in red and in green are respectively referred to as passenger 1 and passenger 2.

3D numerical simulations give unlimited opportunities for studying scenarios of the dissemination of pathogenic biological agents such as the SARS-CoV-2 virus inside a rail coach. In our case, we decided to consider a brief cough and the normal respiration of a passenger assumed to be infected with the virus. In our exploratory computations, the passengers did not wear individual protective masks. The contaminated individual who is coughing was assumed to be seated in compartment 1 of the carriage in one of the two seats shown in Fig. 10. We also took into account a contaminated individual who was breathing and occupying the position of the green manikin in Fig. 10. While arbitrary, both of the chosen locations lead to interesting observations, which are presented later in the results of the dispersion simulations.

Coughing or, alternatively, breathing are typical events causing the dissemination of droplets and drops that are likely to carry the SARS-Cov-2 virus, even more so when the infected individual does not wear a protective face mask. The coughing and breathing were modelled dynamically, meaning that the flow dispersion of the droplets and drops was simulated in an unsteady regime. The details about the dispersion computations are given in the “Methods” section. Essentially, breathing and coughing are both expectorations, but they are very different. In summary, the release of droplets and drops due to a cough is a single event of short duration, and the speed of the air carrying the droplets or drops leaving the mouth is high in comparison with the air velocity around the manikin. On the contrary, the release of droplets due to exhalation is repeated at each cycle of respiration, and the speed of the air carrying the droplets is just slightly higher than the air velocity around the manikin. In the test cases involving a cough, droplets and drops of four different aerodynamic diameters (1, 10, 100 and 1000 µm) were considered, while in the test case involving exhalation, only droplets of 1 µm were taken into account. Furthermore, a realistic number of droplets or drops (in order of magnitude) was released from the mouth of the infected passengers, either 10,000 particles of each size during the cough or 1000 particles for each exhalation.

The three dissemination events reported here (two coughs and one cyclic exhalation) were considered independently. As for the air flow, the transport and dispersion simulations were performed with the CFD model referred to as Code_SATURNE. As far as the dispersion modelling is concerned, two approaches may be employed: Eulerian or Lagrangian. In our study, both approaches were used with two simultaneous aims: first, to compare the results and verify their similarity, and second, to contribute to the development of appropriate methods that are generally applicable to the dissemination of infectious agents in confined, ventilated spaces. In the following sub-section, we present and comment on the results of the dispersion simulations.

### Results of the 3D dispersion simulations corresponding to a cough or to cyclic exhalation

This sub-section is organised in three parts. The first part compares the Eulerian and Lagrangian results for the test case of the brief cough for one location of the infected passenger and micrometric droplets, the second part presents the Lagrangian results for the test case of the brief cough for the other location of the infected passenger and with all sizes of droplets and drops, and the third part presents the Lagrangian results for the test case of the cyclic exhalation with micrometric droplets.

#### Comparison between Eulerian and Lagrangian results

The CFD software operated in this numerical research study offers both the Eulerian and Lagrangian options for the modelling and simulation of the dispersion of aerosols. As there was no reason in principle why one option should be favoured over the other, it was decided to perform the Eulerian and Lagrangian computations together. This sub-section describes the results obtained for the brief cough of passenger 1, who is assumed to emit 10,000 droplets of 1 µm in diameter in 0.5 s.

**Figure 11:**
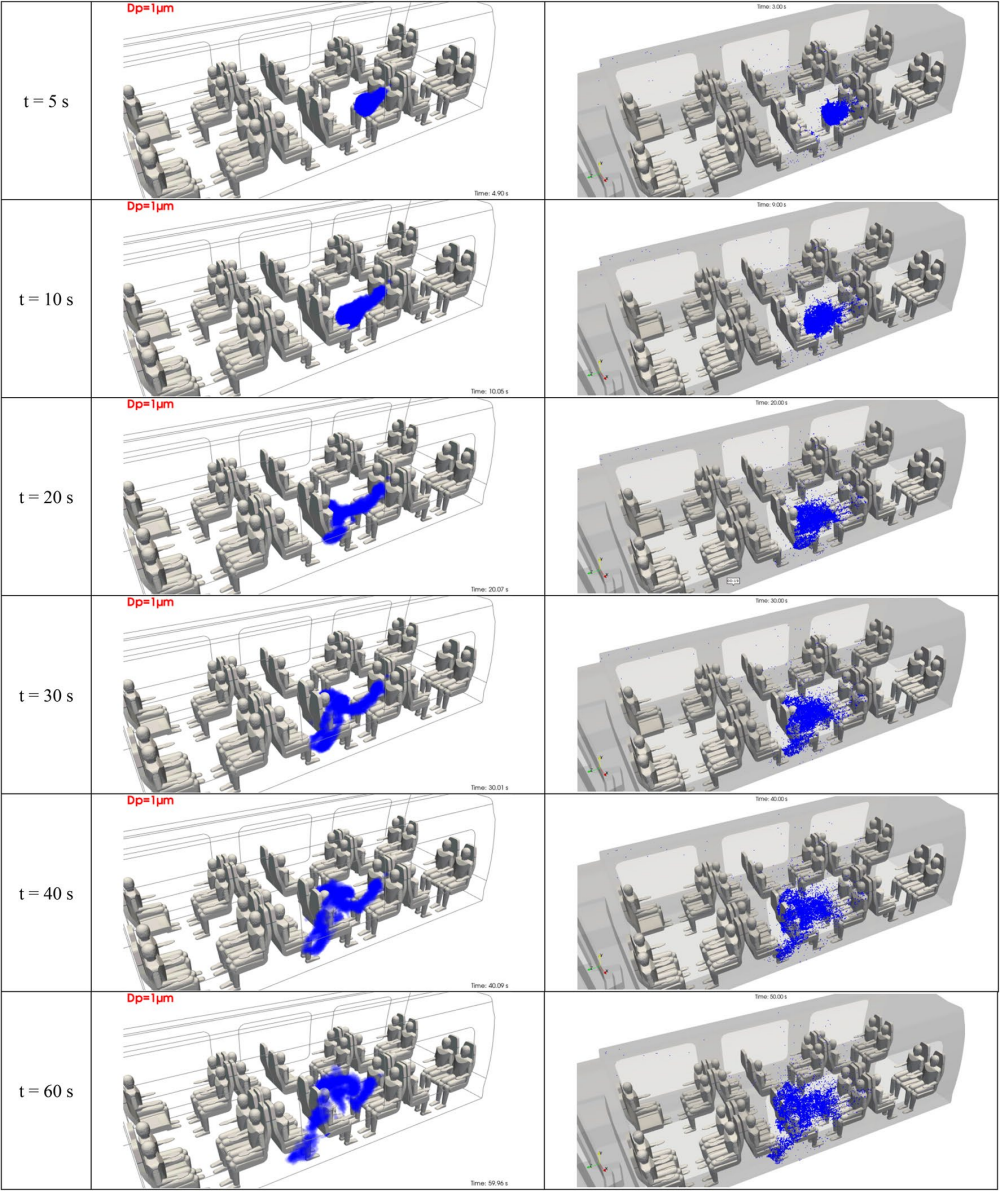
Space and time distribution of the micrometric droplets emitted by passenger 1 after a brief cough. The left and right columns present the dispersion results at the same instants obtained using the Eulerian and the Lagrangian approaches, respectively. The distributions are displayed at 5 s, 10 s, 20 s, 30 s, 40 s and 60 s after the beginning of the cough.

Figure 11 shows the 3D distribution of the micrometric droplets in the inner space of the rail coach. Videos were produced in the framework of this study in order to effectively illustrate the dynamic nature of these results. In this paper, however, we present snapshot views at six successive instants counted after the beginning of the cough. While we have restricted the number of views in order to focus on the first minute of dispersion, results could be provided for longer times.

The Eulerian and Lagrangian approaches are fundamentally different. From the Eulerian point of view, the natural results of the simulations are volumetric concentrations (in droplets per m^3^) obtained by solving a transport and dispersion equation. As this extensive quantity is not very informative regarding the locations of the droplets, it was post-processed to obtain the absolute number of droplets in the cells of the 3D mesh. Next, a surface was created by wrapping the part of the space in the rail coach where the cells contained at least one droplet. This surface is presented in the snapshots in Fig. 11. From the Lagrangian point of view, it is more natural to follow the droplets as each droplet trajectory is solved. The blue dots in Fig. 11 are the locations of all droplets captured for the successive snapshots.

Both the Eulerian and Lagrangian results shown in Fig. 11 lead to the same comments about the distribution of the micrometric droplets generated by passenger 1. From t = 0 to t = 10 s, the droplets experience the effect of the initial impulse given by the cough. They move rapidly in a straight line, perpendicularly with respect to the mouth of the spreader (15° beneath the horizontal direction), towards the passenger seated immediately opposite to the spreader. The plume of droplets also disperses during the same time interval. From t = 10 to t = 20 s, the droplets are in a flow zone between the passengers where the air velocity is very weak, and they tend to stagnate suspended in the air. From t = 20 to t = 60 s, the slow motion and dispersion of the particles operate simultaneously in two directions: vertically, along and around the chest of the passenger opposite to the spreader, and longitudinally, in the direction of the flow ventilating the carriage, towards the lower body of the passenger on the seat row next to the group of seats where the spreader is placed. In the same time interval, the droplets tend to diffuse and dilute in the space between the passengers.

It should be noted that the Eulerian and Lagrangian dispersion results illustrated at six different instants in Fig. 11 are remarkably similar, not only for this test case, but for all situations studied. This is a reassuring output that reinforces the potential conclusions drawn from this research based on numerical simulation.

#### Dynamic behaviour of the droplets and drops generated by a cough

An outstanding feature of the CFD modelling developed in the context of this research is its ability to capture the inherent differences in the aerodynamic behaviour of particles depending on their diameters. Once more, it was verified that the Eulerian and Lagrangian approaches to dispersion led to analogous results and related conclusions. For the sake of concision, only Lagrangian simulations are reported hereafter. This sub-section describes the results obtained for the brief cough of passenger 2, who is assumed to emit 10,000 droplets of either 1, 10, 100 or 1000 µm in diameter, in 0.5 s.

**Figure 12:**
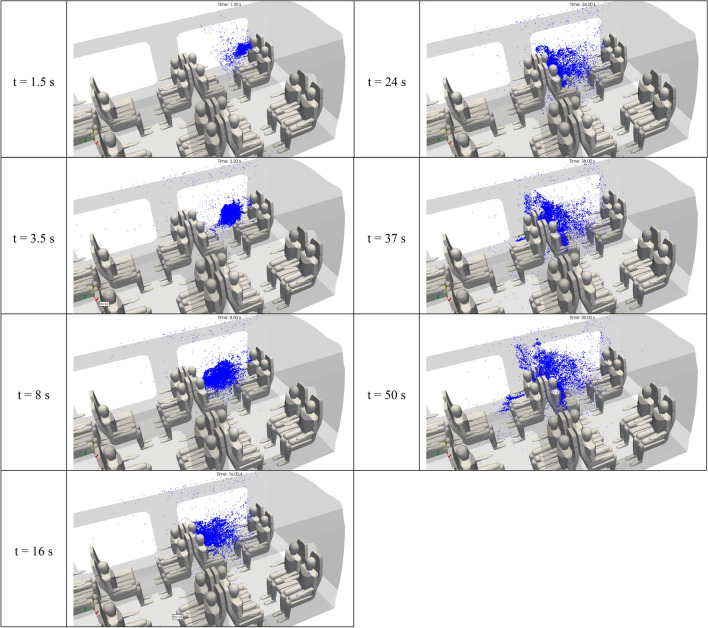
Space and time distribution of the droplets of 1 µm in aerodynamic diameter emitted by passenger 2 after a brief cough. The distributions result from the Lagrangian simulations displayed at 1.5 s, 3.5 s, 8 s, 16 s, 24 s, 37 s and 50 s after the beginning of the cough.

Figure 12 shows the 3D distribution of the droplets of 1 µm in diameter in the inner space of the rail coach. This figure provides snapshot views at seven successive instants from t = 1.5 s to t = 50 s. While it would be possible to show the views made at later times, we have focused on the first minute of dispersion of the micrometric droplets. From t = 0 to t = 10 s, the comments applicable to the droplets released by the passenger 2 are similar to those made for passenger 1 in the previous sub-section. With the initial impulse given by the cough, the droplets move rapidly and perpendicularly to the mouth of the spreader, towards the passenger seated opposite to the spreader. The droplets are projected in a flow zone characterised by very low velocities in the area between the group of four passengers including the spreader. The droplets tend to scatter at a slow pace. After t = 10 s, the micrometric droplets spread on both sides and above the passenger seated opposite to the spreader. They become diluted in the space around this passenger, while also moving along the flow imposed by the ventilation system, and gradually reach the seat row next to the group of four seats including the spreader. With variations compared to coughing passenger 1, the droplets emitted by passenger 2 have more trajectories heading more quickly toward the upper part of the carriage and the extracting vents in the roof. After 1 min of simulated dispersion (results not presented in this paper), the droplets continue moving forward through the carriage with the ventilation flow and being extracted through the vents in the roof. However, they follow complicated swirling trajectories between the seated passengers and around 15 min are necessary to evacuate all droplets.

**Figure 13:**
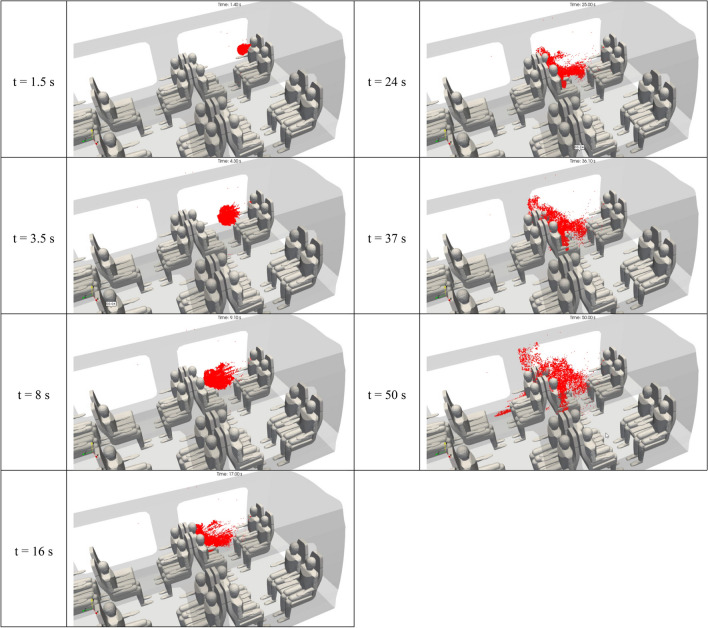
Space and time distribution of the droplets of 10 µm in aerodynamic diameter emitted by passenger 2 after a brief cough. The distributions result from the Lagrangian simulations displayed at 1.5 s, 3.5 s, 8 s, 16 s, 24 s, 37 s and 50 s after the beginning of the cough.

Figure 13 shows the 3D distribution of the droplets of 10 µm in diameter in the inner space of the rail coach. As for the preceding figure, this one provides snapshot views at the same seven successive instants from t = 1.5 s to t = 50 s. The first point to notice is the similar behaviour exhibited by the droplets of 10 µm in diameter compared to the droplets of 1 µm in diameter. Nevertheless, some differences are noticeable. First, the effect of sedimentation is still weak, but it is no longer negligible, and there is a slight drift of the 10 µm droplets from the carrier air flow. Moreover, the spread throughout the rail coach space of the 10 µm droplets is less than the spread of the 1 µm particles. This can be observed at t = 1.5 s, 3.5 s and 8 s, in the cluster formed by the particles located in between the group of four passengers with the spreader. The 10 µm particles tend to gather together, while the 1 µm particles progressively scatter. The more intense scattering of the 1 µm droplets is also visible by means of the blue dots representing the 1 µm droplets in Fig. 12, which are clearly much more numerous and dispersed in a larger part of the carriage space than the red dots standing for the 10 µm droplets in Fig. 13. Furthermore, while the deposition of the 1 µm particles on the accessible surfaces (in particular, the seats and the passengers) is negligible, this is not the case for the 10 µm particles (not shown in this paper).

**Figure 14:**
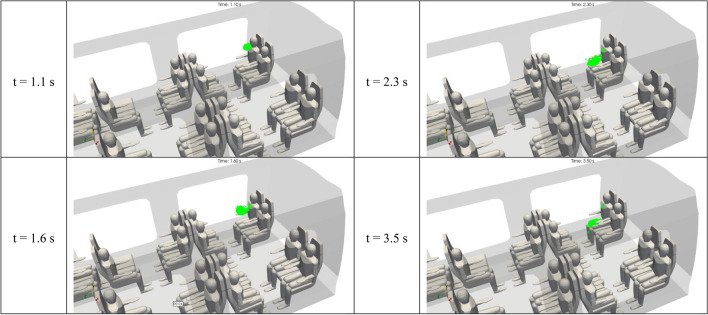
Space and time distribution of the drops of 100 µm in aerodynamic diameter emitted by passenger 2 after a brief cough. The distributions result from the Lagrangian simulations displayed at 1.1 s, 1.6 s, 2.3 s and 3.5 s after the beginning of the cough.

Figure 14 shows the 3D distribution of the drops of 100 µm in diameter in the inner space of the rail coach. This figure provides snapshot views at four successive instants from t = 1.1 s to t = 3.5 s. It is worth noting that these instants are much shorter than those chosen for the droplets of 1 and 10 µm in diameter, indicating that the drops of 100 or 1000 µm in diameter have very different characteristic times. In stark contrast with both Figs. 12, 13 and 14 illustrates the basically distinct aerodynamic behaviour of the 100 µm drops in comparison with the 1 µm and 10 µm droplets. In spite of the initial impulse provided by the cough, the 100 µm drops are subject to gravitational settling and fall down on the knees of the spreader. While not explicitly visible in the figure, the deposition of the 100 µm drops is very effective and happens in the first seconds after the beginning of the cough.

**Figure 15:**
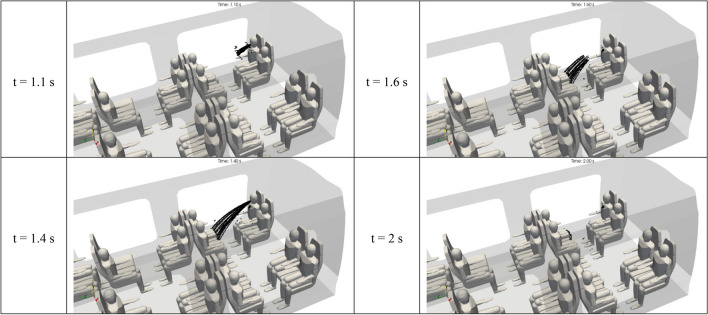
Space and time distribution of the drops of 1000 µm in aerodynamic diameter emitted by passenger 2 after a brief cough. The distributions result from the Lagrangian simulations displayed at 1.1 s, 1.4 s, 1.6 s and 2 s after the beginning of the cough.

Figure 15 shows the 3D distribution of the drops of 1000 µm in diameter in the inner space of the rail coach. This figure provides snapshot views at four successive instants from t = 1.1 s to t = 2 s. Once more, these instants are considerably shorter than those considered for the 1 µm and 10 µm droplets, and even for the 100 µm droplets, revealing extremely different aerodynamic characteristic times. In Fig. 15, the trajectories of the 1000 µm drops are of a ballistic nature. Accounting for the initial impulse due to the passenger’s coughing and the weak velocity of the ambient air flow, the drops are projected like bullets from the mouth of the spreader to the knees of the passenger opposite to the spreader.

In summary, the droplets of 1 or 10 µm in aerodynamic diameter follow the air streamlines perfectly or almost perfectly. In a sense, they are stuck in the flow, adapting to all recirculations and swirling motion without deviating from the streamlines. On the contrary, the drops of 100 or 1000 µm in diameter are subject to inertia and sedimentation effects. Their trajectories cannot follow the streamlines of the air flow and strongly deviate from them. There are also major differences in the aerodynamic behaviour of the 100 and 1000 µm drops, the former being at the limit of what can be called an aerosol, and the latter acting as projectiles should sufficient initial momentum be given to them.

#### Distribution of the droplets emitted in the course of a cyclic exhalation

Expectoration of droplets or drops by individuals possibly infected with the SARS-CoV-2 virus may happen in a variety of ways. In order to extend our research founded on 3D numerical simulation, we also studied the situation of a passenger (#2) placed in the rail coach, breathing normally without a face mask and emitting micrometric droplets. The source term generated by the passenger is very different from the single, brief cough examined above, as in this case respiration is being studied instead. Respiration is a cyclic process including inhalation, exhalation and an interruption of respective approximate durations of 2 s, 2 s and 1 s for an individual at rest. Thus, the source term for breathing is the production of droplets for 2 s every 5 s (which corresponds to 12 respirations per minute). This sub-section describes the results obtained for the calm breathing of passenger 2, who is assumed to emit 1000 droplets of 1 µm in diameter during each of his or her exhalations. The time period considered is relatively long at 585 s; it takes into account 117 exhalations.

**Figure 16:**
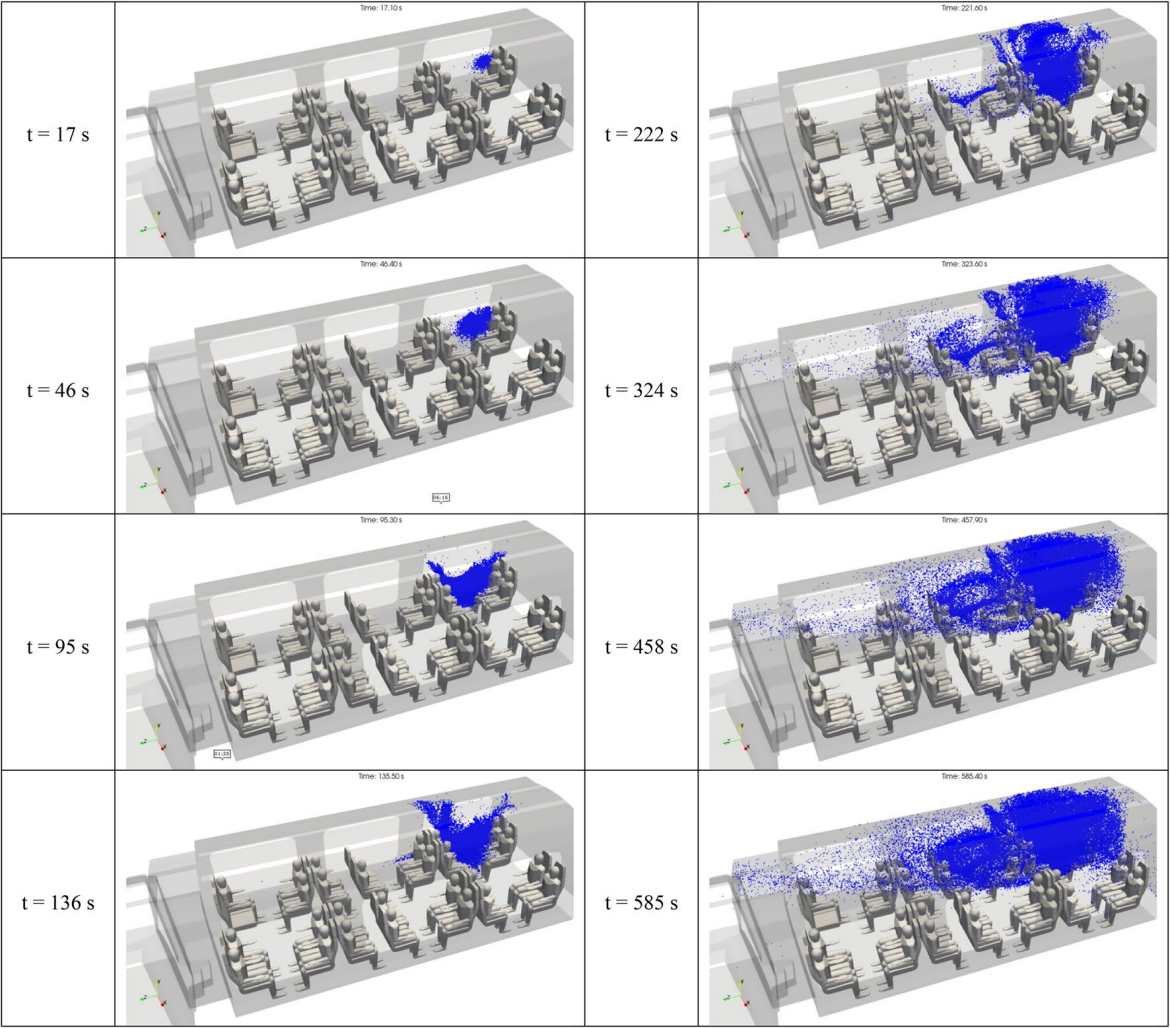
Space and time distribution of the droplets of 1 µm in aerodynamic diameter emitted by passenger 2 during periodic exhalation. The distributions result from the Lagrangian simulations displayed at 17 s, 46 s, 95 s, 136 s, 222 s, 324 s, 458 s and 585 s after the beginning of the breathing cycle.

Figure 16 shows the 3D distribution of the micrometric droplets in the inner space of the rail coach. Once more, such results are dynamic, and a video was produced in the course of the numerical study. In this paper, we present snapshot views at eight successive instants counted after the beginning of the respiration cycle. While we have restricted the number of views and focused on the first ten minutes of the cycle, results could be provided for longer times.

Regarding the fate of the droplets produced by the exhalations of passenger 2, the simulated time period is prolonged by a factor of around 10 in comparison with the cough test cases (passenger 1 or passenger 2). Thus, the transport and dispersion of the droplets is evaluated for much longer durations and distances. One can observe that the micrometric droplets do not stay close to the spreader, but tend to occupy a large part of volume 1 of the rail coach. After about two minutes, the turbulent air flow has both transported and scattered the droplets in between the four passengers closest to the spreader. After about eight minutes, the four passengers next to the first group of passengers on the same side of the carriage are reached by the droplets. While the progression of the droplets occurs principally between and above the passengers seated on the same side as the spreader, some droplets tend to approach the passengers of the seat rows on the other side of the aisle. The droplets are progressively evacuated from the rail coach through the air extraction vents in the roof of compartment 1 of the carriage, or they head towards the extraction vents in the roof of central compartment 2.

The residence time of the droplets in volume 1 of the carriage varies from less than 1 min to more than 10 min. On average, it corresponds to the geometric residence time of the air in the carriage, which is equal to 7 min (see the “Methods” section for more explanations). For a given droplet, the residence time depends on the trajectory. The trajectories of the micrometric droplets are very close to the air flow streamlines, which can be more or less whirling in nature, or can on the contrary go directly from the mouth of the spreader to the air extraction vents. As new droplets are periodically generated by the spreader, the distribution of the droplets in the inner space of the rail coach reaches a stationary regime after a number of cycles of respiration (not shown in the paper).

## Discussion

The research work detailed in this paper is based on physical modelling and numerical simulation using up-to-date CFD. The air flow induced by the ventilation is computed in 3D in the internal space of a public railway transport coach. Moreover, certain passengers seated in the carriage, who are assumed to be infected with the SARS-CoV-2 virus, produce liquid particles of a range of diameters from 1 to 1000 µm when exhaling respiratory air or when coughing. The passengers are not wearing protective face masks, and the small or large particles (respectively called “droplets” or “drops”) are assumed to carry the virus. The transport and dispersion of the particles throughout the carriage are evaluated in 3D, taking account of their size. Other passengers may inhale the particles transported by the droplets or drops and thus be contaminated by the virus.

The main achievements of the numerical study can be summarised as follows:

- Input data have been sought in the relevant literature regarding the geometry and ventilation of suburban trains, and regarding the expectoration of droplets and drops by individuals in the course of processes such as respiration and coughing (as well as speaking, singing or sneezing). Our aim was to reach a satisfying level of realism regarding the internal geometry of the rail coach and the humanoids populating it. Of course, there is still room for improvement in the material and human components of our 3D mock-up.
- The thoroughly validated CFD code referred to as Code_SATURNE has been adapted to model turbulent flow and dispersion in the inner part of a rail coach. The results regarding the flow and dispersion of particles are fully consistent with what could be expected. In particular, the essential differences in the dynamic behaviour of droplets (1 and 10 µm) and drops (100 and 1000 µm) are very well illustrated. While the smaller particles follow the air flow almost perfectly, the larger ones deviate from the air streamlines due to their inertia and their deposition dominated by gravitational settling. Moreover, the dispersion simulations were performed using both the Eulerian and Lagrangian approaches, which showed remarkably similar results.
- The numerical results have been post-processed in order to produce didactic, compelling graphical visualisations, both static (images) and dynamic (videos). Obviously, this is an ancillary, though non-negligible, part of this research, which should be prolonged in practical applications as mentioned later in the paper.

This numerical research has been carried out with the first principal goal of demonstrating the feasibility of properly accounting for the dynamic processes. This method has required many assumptions to be made. As numerous the hypotheses may be, however, there exist opportunities to remove them, offering scientific perspectives for this work that are enumerated hereafter:

- In this study, the air flow turbulence is modelled using a Reynolds-averaged Navier–Stokes k-epsilon model. At the cost of increased computational resources, turbulence could be accounted for using large-eddy simulations. This alternative approach would provide detailed information about the space and time variability of the air flow and processes influencing the distribution of the droplets and drops (mixing, dilution, etc.).
- Mass transfer phenomena such as the evaporation of the droplets and drops or even condensation on dry particles were ignored in the first stage of the simulations. Still, the CFD code used in the study gives modelling possibilities that could be deployed with additional developments. Thus, one could obtain the evolution of the sizes of the droplets and drops when transported and dispersed in the ventilated semi-confined space. Moreover, it could be of interest to take account of the processes of aerosol physics such as nucleation, agglomeration, etc. These supplementary models could use the ambient temperature and relative humidity as parameters, because they seem to have a major effect on the transmission of the virus and on human contamination.
- It would also be a valuable option to model some biological aspects related to the SARS-CoV-2 virus by benefiting from information provided by specialists focusing on this particular virus. For instance, the approximate number of virions in the droplets and drops produced by the infected passenger depending on his or her stage of the disease would be crucial quantitative information for estimating the likelihood of healthy passengers becoming infected. One could also model the depletion of infectivity where appropriate, and more generally the fate of the virus in droplets and drops as they dry up, whether they are suspended in the air or deposited onto accessible surfaces.
- The manikins integrated in the numerical 3D mock-up could be rendered more humanlike and animated. For example, we could alternate manikins of diverse sizes representing male and female adults or children. In this study, we have modelled the mouth of the passenger assumed to be infected with the SARS-CoV-2 virus in order to make him or her breathe out and cough. In the next step, we could easily model the nose of all manikins to make them inhale air and droplets carrying the virus. Furthermore, real spectra of droplets and drops should be used as source terms for the breathing out and coughing. This would be an improvement in the presentation and practical use of the simulations of the dispersion of droplets and drops in enclosed spaces. Finally, other processes leading to the expectoration of droplets or drops, such as speaking or sneezing, could be simulated in order to supplement the results for coughing and breathing out.
- In our study, the infected passenger is not wearing a face mask. In a forthcoming stage of this research work, the manikins could have masks, especially the one who is the spreader. To account for masked manikins in a tractable manner, the method could be to explicitly mesh the bust and face of a sole manikin equipped with a mask, and to simulate the flow field around the manikin when it breathes (inhalation and exhalation) through the mask. Then, the magnitude and direction of the velocity vectors around the mask and the face of the manikin determined from this computation could be used as boundary conditions in the simulations of full semi-confined ventilated spaces such as a rail coach, with a large number of manikins implicitly wearing masks. Another piece of necessary information, which the mask manufacturers often make available, would be the filtration efficiency of the masks as a function of the droplet and drop sizes.

The principal achievement of this research lies in having demonstrated the use of methods associated with a tried and tested computational tool adapted to the 3D simulation of the transport and dispersion of aerosols carrying the SARS-CoV-2 virus or other respiratory viruses. The application of the modelling system to a public railway transport coach has proved useful and relevant. Beyond the stakes of mass transit, the test cases performed can serve as a conceptual illustration of the value of simulations in grasping a complicated phenomenology, and at a further stage could help identify means and measures to limit the dissemination of the SARS-CoV-2 virus.

Following this work, numerous practical perspectives may be envisioned.

- While the studied rail coach is realistic in terms of geometry and ventilation features, it would be of interest for railroad companies to carry out simulations comparable to those presented in the paper using the geometric and HVAC data of real trains and carriages circulating on their networks. In particular, the actual operation of an air conditioning system mostly involves partial recycling of the air passing through the carriage. This should be considered using data on the air fraction, the filters and, possibly, the air purification systems in place on a case-by-case basis, beyond the scope of this generic study. Furthermore, this would be very useful and important for studying the effects of a modified internal carriage configuration on the air flow and distribution of the liquid particles expectorated by passengers. For instance, the modifications could consist in a reversal of the seats by positioning them in front of the windows or, more simply, the mounting of partitions between some of the seat rows. Numerical simulations are cheaper and easier to carry out than experiments, and they can provide precious information about the configurations that are most able to limit the dissemination of the droplets.
- As mentioned before, the concepts underlying our numerical study can be applied to any kind of ventilated, more or less confined, large or small, private or collective-use space. Thus, utilising the same methods and computational tool, one could proceed with the simulation of SARS-CoV-2 dissemination in means of transport such as planes or cruise ships, or in multiple places such as restaurants, performance halls, nurseries, classrooms, open-plan offices, factories, workshops or slaughterhouses. Moreover, CFD numerical studies of semi-open spaces, such as the bleachers in a stadium or the narrow streets of densely built urban districts in historic or tourist destinations, could be undertaken according to the principles and tools developed in this paper.
- Subsequently, the 3D numerical simulations of SARS-CoV-2 dissemination in the form of aerosols should be extremely valuable for estimating the criticality of the gathering of people, when some of them are infected with the virus in closed or semi-closed spaces such as those given as examples above. General guidance and recommendations could be deduced, thereby contributing to tackling the SARS-CoV-2 virus or other respiratory viruses.

## Methods

The central tenet of this numerical research is to exploit a proven, reliable CFD tool to replace experiments in the real world. This strategy is appropriate insofar as the computational tool operated in the study has been thoroughly validated for simulations of the dispersion of aerosols in laminar or turbulent flows. This study is based on a series of stages comprising choices related to the modelling, the search for available relevant data, the development of the 3D mock-up of the ventilated space occupied by human beings, the implementation of flow and dispersion simulations and, finally, the post-processing of the results to make them easily exploitable. The choices for the physical modelling mainly relate to the turbulence of the flow, the dispersion of the droplets or drops and their deposition onto the accessible surfaces (rail coach walls, seats, passengers, etc.). The choices for the numerical modelling relate to the type and other characteristics of the meshing and to numerical parameters such as the time step. Otherwise, a number of data are necessary to run the simulations. The data principally relate to the geometry and ventilation of the rail coach, the occupancy of the rail coach by the passengers, and the characteristics of the droplets and drops generated by the passengers. Some of these modelling and data aspects are reviewed in the following part of this section.

### Modelling with Code_SATURNE

All of the simulations presented in this paper were carried out using Code_SATURNE, a general-purpose, open-source CFD computational tool developed by the R&D division of the French electricity supplier EDF and by the Atmospheric Environment Teaching and Research Centre (CEREA) in Paris, France. Code_SATURNE is a finite volume code using a fully co-located arrangement for all variables on structured or unstructured 2D or 3D meshes with various kinds of cells. It has many numerical solvers, mainly based on prediction and correction steps, for laminar or turbulent, steady or unsteady, uncompressible or compressible, isothermal or non-isothermal, non-reactive or reactive, monophasic or multiphasic flows.

Code_SATURNE implements several approaches for turbulence modelling in the Reynolds-averaged Navier–Stokes and large-eddy simulations formalisms. In this study, we have chosen to use the former approach and, more precisely, the standard k-epsilon model. This turbulence model has known limitations related in particular to curvature, density stratification or swirl if these physical phenomena are present^36^, which is not the case of the numerical study in the carriage. Furthermore, the k-epsilon model is known as robust, well adapted to moderate Reynolds number turbulent ventilation flows, and provides results in quite low amounts of time. It is thus widely used for scientific and industrial applications^37^. As proven by the convincing results of the numerical study, this turbulence approach seems adequate to depict the average transport and dispersion of droplets or drops carried by the air flow. In further development, however, large-eddy simulations could be considered, because they would indeed provide more information about the 3D space and time fluctuations of the turbulent flow acting on the turbulent dispersion of the droplets and drops.

As for dispersion modelling, Code_SATURNE has the great advantage of proposing both the Eulerian and Lagrangian approaches. In the former, solid or liquid particles are treated as a phase carried by the air flow, and their volumetric concentration is obtained by solving a transport and dispersion equation (if the particles have different diameters, they can be sorted by classes with as many equations solved as classes of particles). In the latter, solid or liquid particles are considered individually, and their trajectories are determined by solving as many equations of motion as there are particles. The Eulerian approach is deemed to be subject to numerical diffusion, though this drawback may be limited by relevant choices of numerical schemes. The Lagrangian approach is more complex to utilise, but also the more appropriate method when it comes to accounting for all forces acting on the particles. Both approaches take into account the average and fluctuating components of the flow field, the latter component being evaluated on the basis of the turbulent properties of the flow.

### Equations in Code_SATURNE

For the sake of completeness, the equations solved by Code_SATURNE in the numerical study are presented below starting with the turbulent flow model, then the Eulerian and the Lagrangian dispersion models.

In RANS formalism, the continuity and momentum equations of an incompressible flow are written as follows:

$$\frac{\partial \rho }{\partial t}+\frac{\partial }{\partial {x}_{i}}\left(\rho {u}_{i}\right)=0$$

$$\frac{\partial }{\partial t}\left(\rho {u}_{i}\right)+\frac{\partial }{\partial {x}_{j}}\left(\rho {u}_{i}{u}_{j}\right)=-\frac{\partial p}{\partial {x}_{i}}+\frac{\partial }{\partial {x}_{j}}\left[\mu \left(\frac{\partial {u}_{i}}{\partial {x}_{j}}+\frac{\partial {u}_{j}}{\partial {x}_{i}}-\frac{2}{3}{\delta }_{ij}\frac{\partial {u}_{l}}{\partial {x}_{l}}\right)\right]+\frac{\partial }{\partial {x}_{j}}\left(-\rho \overline{{u_{i}^{\prime}} {u_{j}^{\prime}}}\right)$$

with the usual notations of the mathematical operators, $t$ the time, ${x}_{i}$ (i = 1, 2, 3) the space coordinates, $\rho$ the fluid density, $p$ the pressure, u the mean velocity and u’ the fluctuating velocity. In eddy viscosity models like the standard $k$–$\varepsilon$ model, the Reynolds stresses $-\rho \overline{{u_{i}^{\prime}} {u_{j}^{\prime}}}$ are related to the mean velocity gradients using the turbulent viscosity ${\mu }_{T}$ assumed as an isotropic scalar quantity:

$$-\rho \overline{{{u }^{{\prime}}}_{i}{{u}^{{\prime}}}_{j}}= {\mu }_{T}\left(\frac{\partial {u}_{i}}{\partial {x}_{j}}+\frac{\partial {u}_{j}}{\partial {x}_{i}}\right)-\frac{2}{3}\left(\rho k+{\mu }_{T}\frac{\partial {u}_{k}}{\partial {x}_{k}}\right){\delta }_{ij}$$

Two transport equations for the turbulent kinetic energy $k$ and the turbulence dissipation rate $\varepsilon$ are solved:

$$\frac{\partial }{\partial t}\left(\rho k\right)+\frac{\partial }{\partial {x}_{i}}\left(\rho {ku}_{i}\right)=\frac{\partial }{\partial {x}_{j}}\left[\left(\mu +\frac{{\mu }_{T}}{{\sigma }_{k}}\right)\frac{\partial k}{\partial {x}_{j}}\right]+{G}_{k}+{G}_{b}-\rho \varepsilon$$

$$\frac{\partial }{\partial t}\left(\rho \varepsilon \right)+\frac{\partial }{\partial {x}_{i}}\left(\rho {\varepsilon u}_{i}\right)=\frac{\partial }{\partial {x}_{j}}\left[\left(\mu +\frac{{\mu }_{T}}{{\sigma }_{\varepsilon }}\right)\frac{\partial \varepsilon }{\partial {x}_{j}}\right]+{C}_{1\varepsilon }\frac{\varepsilon }{k}\left({G}_{k}+{C}_{3\varepsilon }{G}_{b}\right)-{C}_{2\varepsilon }\rho \frac{{\varepsilon }^{2}}{k}$$

where ${G}_{k}$ and ${G}_{b}$ represent the generation of turbulent kinetic energy due to respectively mean velocity gradients and buoyancy (${G}_{b}$ is ignored in our study); ${C}_{1\varepsilon }$, ${C}_{2\varepsilon }$ and ${C}_{3\varepsilon }$ are empirical constants, and ${\sigma }_{k}$, ${\sigma }_{\varepsilon }$ the turbulent Prandtl numbers for $k$ and $\varepsilon$. The turbulent viscosity ${\mu }_{T}$ is computed as a function of $k$ and $\varepsilon$: ${\mu }_{T}=\rho {C}_{\mu }\frac{{k}^{2}}{\varepsilon }$ where ${C}_{\mu }$ is a constant. More details about the $k$–$\varepsilon$ model and the values of the standard constants can be found in the seminal work by Launder and Spalding^38^.

Near the walls, a special interpolation method is needed since *k* and $\varepsilon$ take large values compared to the core of the flow and the linear interpolation method used by a finite volume solver like Code_SATURNE is no longer appropriate. In the study, an enhanced near walls treatment was implemented through a scalable wall function (also known as hybrid wall function) for modelling of the laminar sub-layer and buffer region throughout the turbulent flow simulation. This approach is a blended wall treatment for moderate Reynolds number flows and $k$–$\varepsilon$ based CFD simulations depending on the local value of the dimensionless wall distance ${y}^{+}$. This approach is known to avoid issues in CFD problems with different velocity scales on a rather uniform near-wall mesh. In this study the ${y}^{+}$ map around the manikins and inside the carriage highlights a very good resolution with ${y}^{+}$ values between 1 and 80 near the mouth of the manikins.

The Eulerian dispersion model lies on the advection–diffusion equation of the particles written like this:

$$\frac{\partial C}{\partial t}+\frac{\partial }{\partial xi}\left[\left({u}_{i}+{V}_{s,i}\right)C\right]=(\frac{\mu }{Sc}+\frac{{\mu }_{T}}{S{c}_{T}})\frac{\partial ^2 C}{\partial xi^2}$$

with $C$ the concentration (in particles per m^3^), $u$ the mean fluid velocity, ${V}_{s}$ the settling velocity, and $Sc$, $S{c}_{T}$ respectively the molecular and the turbulent Schmidt numbers. The settling velocity is expressed as ${V}_{s}={\tau }_{p}.g$ where $g$ stands for the gravity and ${\tau }_{p}$ is the particle relaxation time, *i.e.* the time needed by the particles to adjust their velocity to the flow velocity. The deposition of the particles on accessible surfaces is considered using Lai and Nazaroff model^39^ whose details are out of the scope of this article.

The Lagrangian dispersion model lies on the implementation of the Langevin stochastic equation developed by Minier and Peirano^40^. As the volume fraction of the particles is small compared to that of the fluid, one-way coupling is considered, which means that the action of the particles on the flow can be neglected. The following set of equations is solved for each particle with a position ${x}_{p}$ and a velocity ${U}_{p}$:

$$\left\{\begin{array}{l}d{x}_{p,i}(t)={U}_{p,i}dt\\ d{U}_{p,i}\left(t\right)=\frac{{U}_{s,i}-{U}_{p,i}}{{\tau }_{p}}dt+{g}_{i}dt\\ d{U}_{s,i}\left(t\right)={A}_{s,i}dt+{A}_{p\to s,i}dt+{B}_{s,ij}d{W}_{j}(t)\end{array}\right.$$

where ${U}_{s}$ is the fluid velocity seen by the particle which requires to be modelled, ${A}_{s}$ the drift vector, ${A}_{p\to s,i}$ the acceleration of the particle, and ${B}_{s}$ the diffusion matrix, which are defined by the following formulae:

$${A}_{s,i}=-\frac{1}{\rho }\frac{\partial p}{\partial {x}_{i}}+\left({U}_{p,j}-{u}_{j}\right)\frac{\partial {u}_{i}}{\partial {x}_{j}}-\frac{1}{{T}_{L,i}^{*}}\left({U}_{s,i}-{u}_{i}\right)$$

$${B}_{s,i}^{2}=\varepsilon \left({C}_{0}{b}_{i}\tilde{k }/k+ \frac{2}{3}\left(\frac{{b}_{i}\tilde{k }}{k}-1\right)\right)$$

$${A_{p \to s,i}} = {{\left( {{U_{p,i}} - {U_{S,i}}} \right)}}/{{{\tau _p}}}\quad T_{L,i}^ * = \frac{{{T_L}}}{{\sqrt {1 + \beta _i^2\frac{{{{\left| {{U_p} - {U_s}} \right|}^2}}}{{\frac{{2k}}{3}}}} }}\quad {b_i} = \frac{{{T_L}}}{{T_{L,i}^ * }}\quad \tilde k = \frac{3}{2}\frac{{\sum\nolimits_{i = 1}^3 {{b_i}} u_i^2}}{{\sum\nolimits_{i = 1}^3 {{b_i}} }}$$

where ${\beta }_{i}$ and ${C}_{0}$ are constants, ${T}_{L}$ is the Lagrangian time scale and dW_i_ the increment of a Wiener process. The relaxation time ${\tau }_{P}$ needed by the particle to adjust its velocity to the flow velocity is defined by:

$${\tau }_{P}=\frac{{\rho }_{P}}{\rho }\frac{4{d}_{p}}{3{C}_{D}\left|{U}_{p}-{U}_{s}\right|}$$

with ${d}_{p}$ the aerodynamic diameter of the particle, and ${C}_{D}$ the drag coefficient computed from the relation:

$${C}_{D}=\left\{\begin{array}{l}\frac{24}{{Re}_{p}}\left(1+0.15{Re}_{p}^{0.687}\right)\quad if \quad {Re}_{p}<1000\\ 0.44 \quad \quad \quad if\quad {Re}_{p}>1000\end{array}\right.$$

where ${Re}_{p}$ is the Reynolds number of the particle defined by $\left|{U}_{p}-{U}_{s}\right|{d}_{p}/\nu$ with $\nu$ the kinematic viscosity of the fluid.

### Numerical schemes and options in Code_SATURNE

Regarding the setup of Code_SATURNE, the SIMPLEC velocity–pressure coupling algorithm was implemented in all simulations, which were carried out in two stages. In the first stage, the steady-state flow generated by the ventilation system in the rail coach was computed. For this purpose, a pseudo-steady CFL-limited solver with a space- and time-varying time step was used. In the second stage, the flow, which was locally perturbed by a dissemination event (a cough or breathing out), was computed again along with the dispersion of droplets and drops, using either an Eulerian approach or a Lagrangian approach. The Eulerian approach consisted in solving, in addition to the flow, the transport equation of the volumetric concentration of the droplets or drops. A solver with an adaptive time step and a reference time step of 0.1 s was used. The Lagrangian approach consisted in solving the dynamic equations of individual droplets or drops. A constant time step was used; it was shorter for the faster event (the cough) and longer for the slower event (breathing). This parameter was crucial for the accuracy of the results, and it was chosen carefully depending on the mesh spatial discretisation. Tests showed that time steps of 0.005 s for the cough and 0.1 s for the breathing were suitable.

### Validation of Code_SATURNE

Code_SATURNE has been developed by EDF under quality assurance and is used both in industry and for academic research. It is applied to various fields such as aeraulics (heating, ventilation and air conditioning, pollutant dispersion), engines (turbomachinery), combustion (gas and coal furnaces) and process engineering (plasma, electric arc, glass furnace). Efforts were invested into qualification processes in several areas, like for instance nuclear thermal-hydraulics. Code_SATURNE was validated against measurements in a large number of academic case studies exhibiting analytical solutions and industrial applications implying complex geometries^41^. To give a few examples of validation files by restricting to internal flows and the closest configurations to the numerical study presented in this article, let us mention the ventilation of premises comprising several rooms possibly undergoing fire, the distribution of chemical species in a gas turbine, and the trajectories and deposition of pulverized coal particles in a furnace. All the case studies corresponded to turbulent regimes and were based on the resolution of the Navier–Stokes equations in RANS formalism using the k-epsilon turbulence model. In the last two cited studies, the Eulerian transport equation and the Lagrangian dynamics equations of particles in size ranges from a few microns to a few tens of micrometers were solved respectively.

It is worth mentioning that Code_SATURNE was also validated in a case study corresponding to a hospital room intended for intensive care of major burns^42^. This type of patient is particularly sensitive to infectious agents (bacteria or fungal spores) in the form of particles suspended in the air. The calculation domain included the treatment chamber accessed from two anti-chambers, as well as the patient and medical staff. The flows were generated by forced convection (ventilation system) and free convection (due to temperature differences between rooms). Potential sources, emitting particles 1 to 20 µm in aerodynamic diameter, were placed at different points in the calculation domain. The simulations consisted in solving the Navier–Stokes equations and two transport equations for temperature and particles in RANS formalism. Turbulence model was the standard k-epsilon. The turbulent Prandtl and Schmidt numbers were taken equal to one. Both the stationary case (RANS) with the doors of the different rooms closed and the unsteady case (URANS) with the doors between the different rooms first opened, then closed (to generate the transfer of particles between rooms) were considered. The numerical results of Code_SATURNE were compared to experimental results obtained in the same geometry and for the same conditions of ventilation and heating of the premises. The agreement was very satisfactory for both the velocity field and the particle concentration field. The validation of Code_SATURNE in this specific case study reinforces our use of the software in the numerical study of the railroad carriage, in which the same turbulent flow model and Eulerian dispersion model were implemented.

The Lagrangian dispersion model in Code_SATURNE also benefited from a great attention. The general principles were established for turbulent two-phase flows carrying particles, which might be bubbles, drops or solid inclusions^40,43^. In many cases, the carrier fluid phase is treated in RANS formalism, in particular with k-epsilon turbulence model. The carried phase, that is to say the particles, are taken into account by considering, on the one hand their dynamics where drag and gravity are generally the preponderant forces, on the other hand the stochastic equation of Langevin which shows relaxation and drift terms as well as a random term in the form of a Wiener process. When the particle size is small compared to the scales of turbulence or the volume fraction of the particles is small compared to that of the fluid, the action of the particles on the flow can be ignored. The Lagrangian dispersion model was validated for various turbulent flows transporting particles with aerodynamic diameters between 1 and 100 µm in pipes, above walls, in jets as well as in a bluff-body configuration where recirculation occurs^44^. In this set of cases, the numerical results were close to the experimental results as can be seen in detail in the mentioned article. Finally, it is interesting to note that studies were carried out to ensure the consistency between the Eulerian approach to turbulent flow and the Lagrangian statistical approach to particle dispersion^45^. It was shown that coherence is ensured by checking in particular the well-mixed condition which is obtained by taking the average pressure gradient as the drift term in the Langevin equation, as is the case in Code_SATURNE.

### Details about the ventilation of the rail coach

Determining the features of the ventilation system was an important step in the numerical study. The data relate to the conditions of air supply and extraction, that is to say the location, shape and dimensions of the supply and extraction vents and the flow rates through these vents. The data chosen in our simulations were inspired by a review of air conditioning and ventilation in trains, and by the geometry files of the rail coach taken as an example.

**Figure 17:**
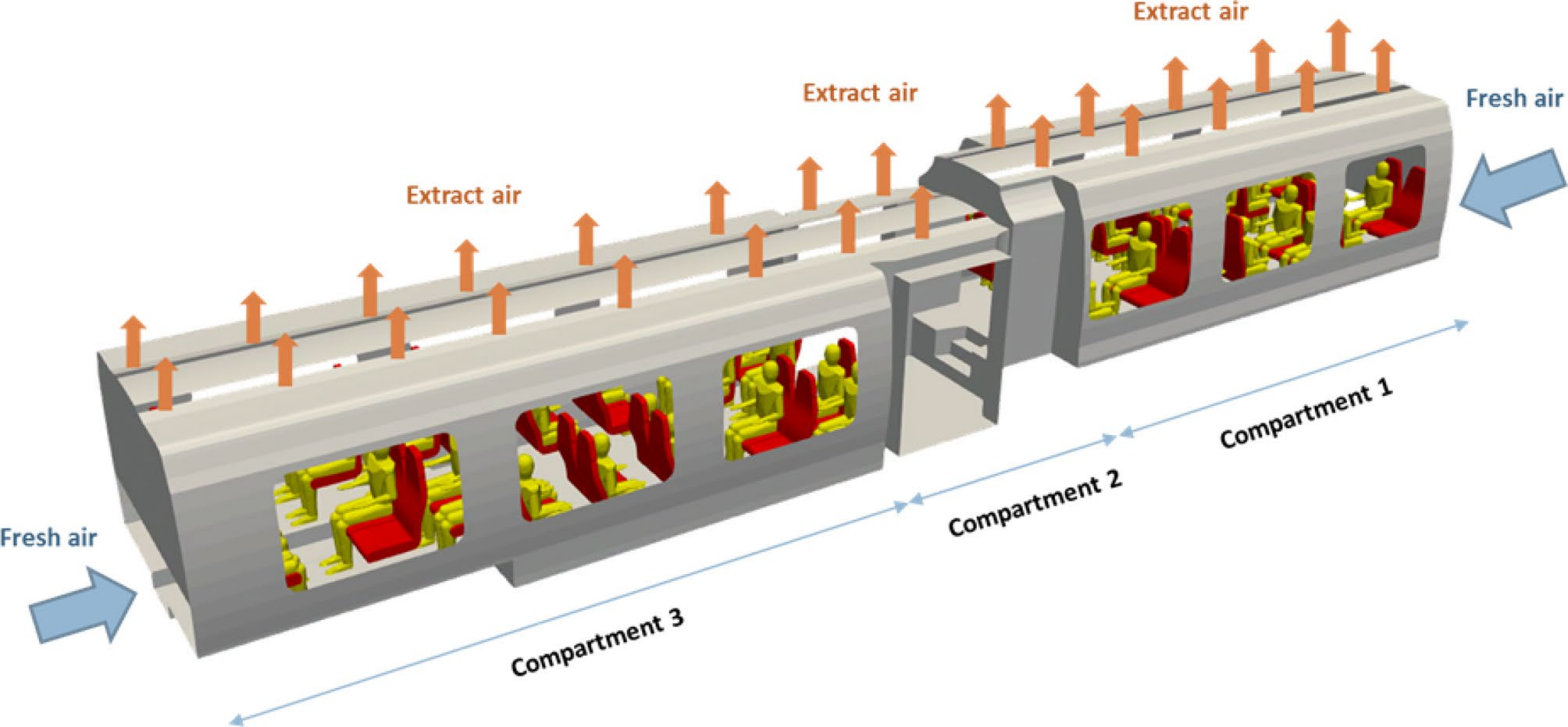
Layout of the ventilation in the CFD mock-up of the rail coach. The air is blown in at the ends of the carriage and is extracted through splits in the roof of the carriage. Compartments 1 and 3 are occupied by the passengers. Compartment 2 is the place where they access the rail coach.

Figure 17 sketches the layout of the ventilation in the rail coach. The flow rates through the extracting vents in compartments 1 and 3 were adjusted to generate a flow motion from the ends to the middle of the rail coach and to provide for the extraction of 50% of the air through compartment 2.

Table 1 presents the data relating to the ventilation of the carriage. The whole velocity of the supplied or extracted air is less than 0.2 m s^−1^. The air renewal rate in each compartment of the carriage is computed using the flow rate supplied to this compartment. It is equal to 8.7. The residence time is obtained by dividing the volume of each compartment by the air flow rate supplied to this compartment. It represents the average residence time of the air in this compartment.

**Table 1:** Ventilation characteristics of the compartments occupied by the passengers.

	Compartment 1	Compartment 3
Volume	27.34 m^3^	30.49 m^3^
Supplied flow rate	0.066 m^3^ s^−1^	0.074 m^3^ s^−1^
Extracted flow rate	0.033 m^3^ s^−1^	0.037 m^3^ s^−1^
Extraction deficit	0.033 m^3^ s^−1^	0.037 m^3^ s^−1^
Air renewal rate	8.7	8.7
Residence time	414 s	412 s

These data were used in the simulation of the air flow in the inner space of the rail coach.

### Sensitivity of the flow results to the mesh refinement

Code_SATURNE simulations of the flow generated by the ventilation in the rail coach were carried out using unstructured CFD meshes of tetrahedral cells. Three meshes with different geometrical characteristics and level of refinement were benchmarked. For all meshes, the minimum cell size was 1 cm near the mouth of the manikins and 3 cm on the body of the manikins and on the seats. Mesh #1 (respectively #2 and #3) had 16 (respectively 11 and 4) million cells of maximum size 5 (respectively 5 and 10) centimetres and a cell size progression factor of 10% (respectively 20% and 20%). The flow results were compared in order to evaluate the sensitivity of the numerical solution as a function of the mesh refinement. This analysis showed that the flow fields were almost identical with the finest, intermediate, and lightest meshes (respectively 16, 11, and 4 million cells) in all compartments of the carriage, in particular in the two compartments of interest occupied by the human manikins. Based on this observation, the lightest mesh was advantageously used to run the flow as the Eulerian and Lagrangian dispersion simulations.

### Dissemination events involving droplets or drops

The dissemination events considered in the numerical study originate either from a cough or from exhalation. While in both cases droplets or drops are expectorated by the passenger, these events are associated with distinct source terms. The cough leads to a single brief release. Of course, more than one cough, as occurs with a coughing attack, could be considered, with several coughs simulated one after the other. In contrast, exhalation leads to a periodic release related to the respiration cycle. The initial impulse of the expectoration is much higher for the cough than for the exhalation. Yet, in either case, the impulse is directed orthogonally to the mouth of the passenger, with an angle of 15° beneath the horizontal direction. Another difference between coughing and exhalation is the size of the particles produced. While coughing may lead to a full spectrum of droplets and drops, breathing out produces micrometric droplets. Regarding the cough, we decided to consider particles separately over a wide range of sizes, from 1 to 1000 µm in aerodynamic diameter. In a further stage of this research, it would be interesting to adopt a more realistic spectrum produced in the event of a cough. It is also worth noting that other dissemination events such as sneezing or speaking could be envisaged. Indeed, these events are quite close to coughing and breathing out, respectively. Tables 2 and 3 compile the features of the cough and the exhalation, respectively, which were considered when constituting the source terms in the dispersion simulations.

**Table 2:** Characteristics of a cough disseminating droplets and drops of different sizes.

Type of dissemination event	A cough is a single expectoration from the mouth
Duration of coughing	0.5 s
Velocity of the expectorated air	4.5 m s^−1^
Direction of the expectorated air	Perpendicular to the mouth, 15° beneath the horizontal direction
Aerodynamic diameters of the particles	4 classes: 1 µm, 10 µm, 100 µm and 1000 µm
Number of particles	10,000 in each class of particles

**Table 3:** Characteristics of the exhalation disseminating micrometric droplets.

Type of dissemination event	Breathing out is an intermittent expectoration from the mouth
Respiratory frequency	12 respirations per minute, 5 s per cycle of inhalation/exhalation/break
Duration of the exhalation	2 s (2/5 of a respiratory cycle)
Velocity of the expectorated air	0.2 m s^−1^
Direction of the expectorated air	Perpendicular to the mouth, 15° beneath he horizontal direction
Aerodynamic diameter of the particles	1 class: 1 µm
Number of particles	1000 for each exhalation

### Computational resources and computational times

The 3D numerical study was carried out using a workstation with a Bi-Xeon® Intel CPU processor equipped with 2 × 16 hyper-threaded cores. The characteristics of the steady flow and unsteady dispersion simulations associated with the dissemination events are given in Table 4 with the duration of the simulations as the prominent information.

**Table 4:** Main features of the aeraulics and dispersion of the droplets and drops considered in the simulations.

Characteristics	Steady flow computation	Cough		Breathing out
		Eulerian unsteady dispersion computation	Lagrangian unsteady dispersion computation	Lagrangian unsteady dispersion computation
Number of mesh cells	4 million			
Number of cores	1	10	15	20
Time step	–	From 0.01 to 0.2 s	0.01 s	0.1 s
Simulated physical time	–	475 s	50 s	600 s
Computation duration	12.4 h	22.2 h	71.6 h	71.4 h

## Data Availability

All graphical results, both static and dynamic, are available from the authors upon request.
